# Supplementary figures and images for: Subfunctionalized expression drives evolutionary retention of ribosomal protein paralogs Rps27 and Rps27l in vertebrates
Source: eLife. 2023 Jun 12;12:e78695. doi: 10.7554/eLife.78695 (PMC10313321; doi:10.7554/eLife.78695)

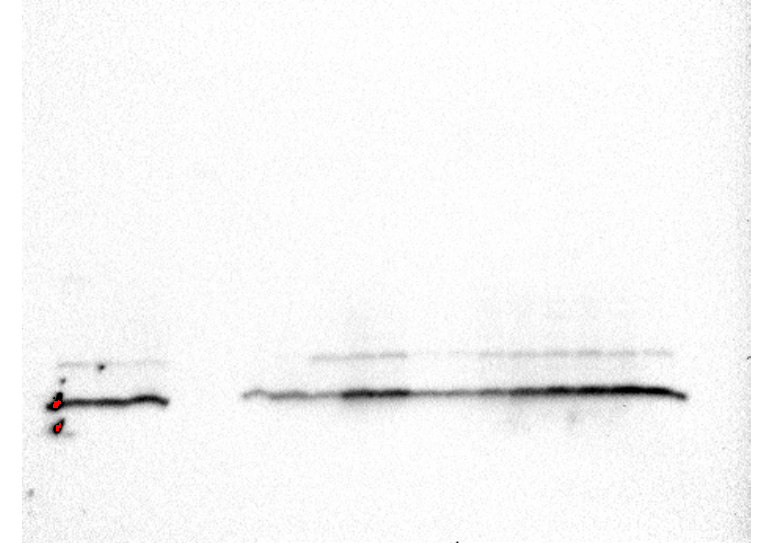

Supplement: Figure 3—source data 1. [file elife-78695-fig3-data1.zip › Figure 3--Source Data 1--20180717 GFP grad S27 WB 382.1 sec.png]

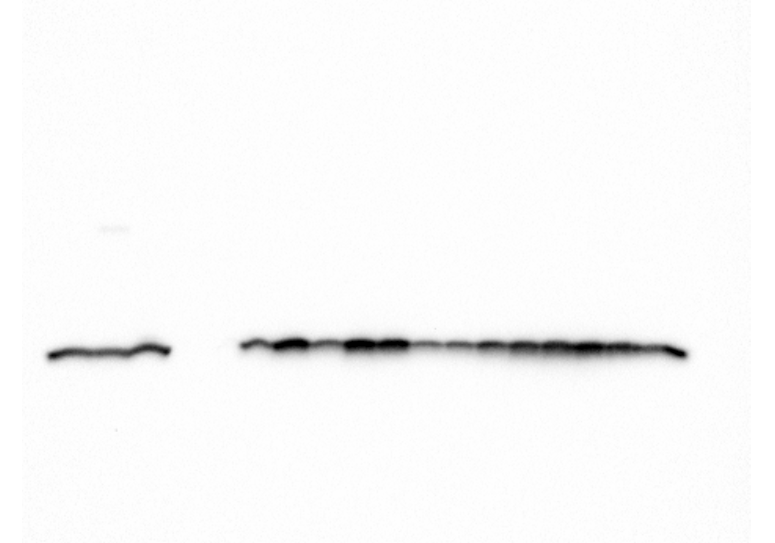

Supplement: Figure 3—source data 2. [file elife-78695-fig3-data2.zip › Figure 3--Source Data 2--20180717 GFP grad S27L PT WB 13.1 sec.png]

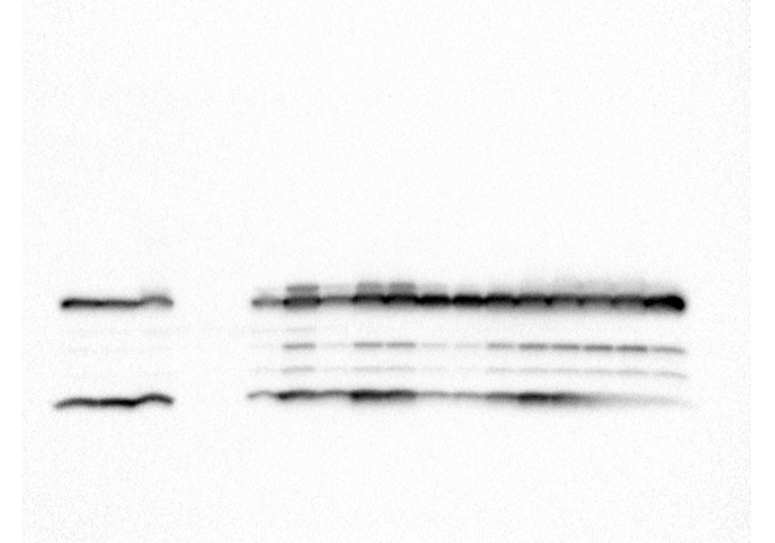

Supplement: Figure 3—source data 3. [file elife-78695-fig3-data3.zip › Figure 3--Source Data 3--20180720 GFP grad S27L reblot S3-A303-840A WB 41.6 sec.png]

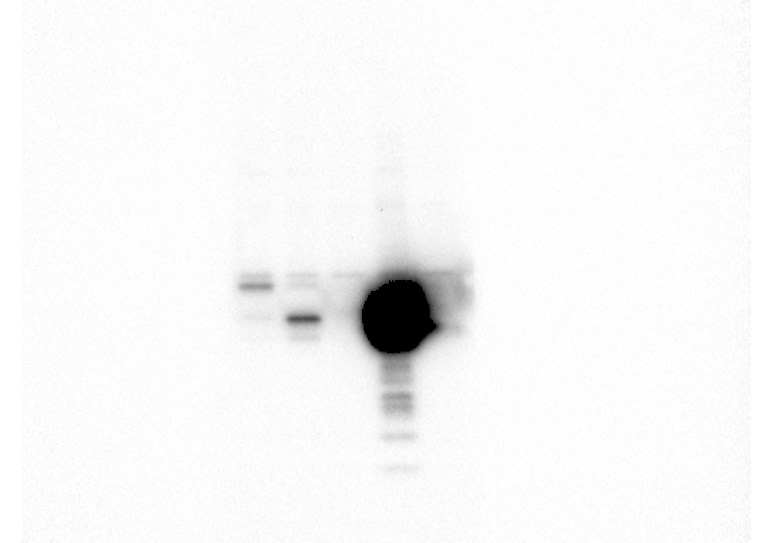

Supplement: Figure 3—figure supplement 1—source data 1. [file elife-78695-fig3-figsupp1-data1.zip › Figure 3-figure supplement 1--Source Data 1--20180716 S27L-GFP-FLAG FLAG redo 103.8 sec no red.png]

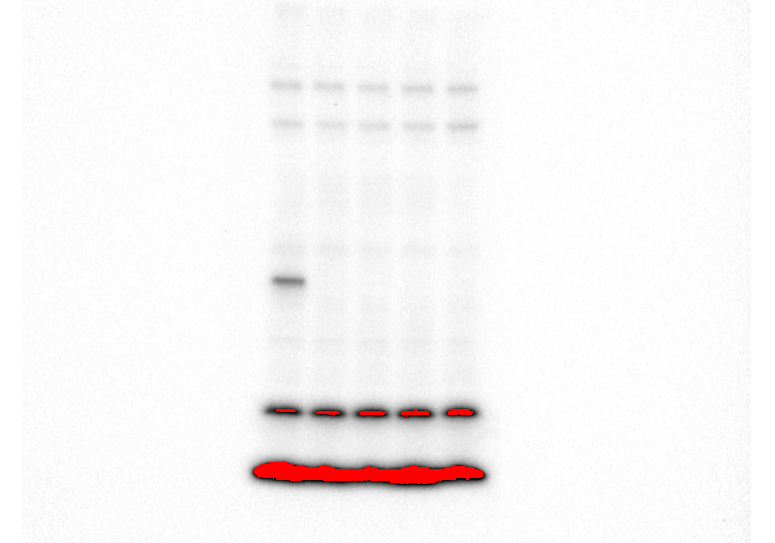

Supplement: Figure 3—figure supplement 1—source data 2. [file elife-78695-fig3-figsupp1-data2.zip › Figure 3-figure supplement 1--Source Data 2--20180713 S27L-GFP-FLAG S27 Pierce 147.4 sec.png]

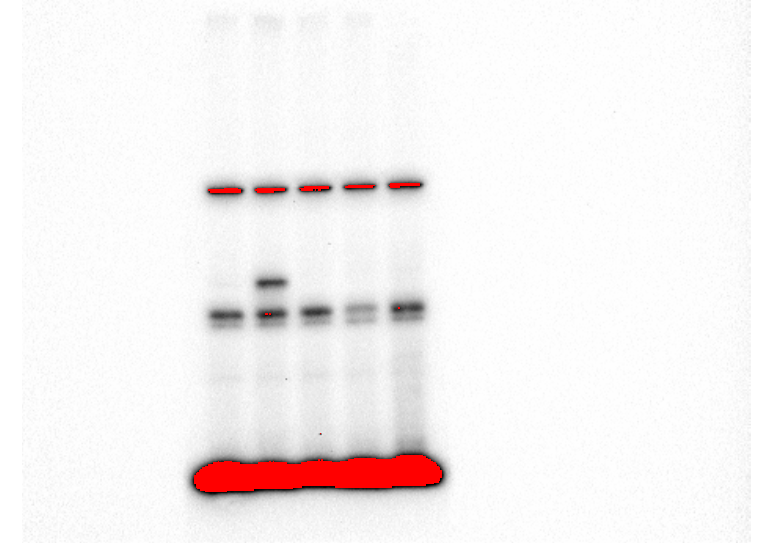

Supplement: Figure 3—figure supplement 1—source data 3. [file elife-78695-fig3-figsupp1-data3.zip › Figure 3-figure supplement 1--Source Data 3.png]

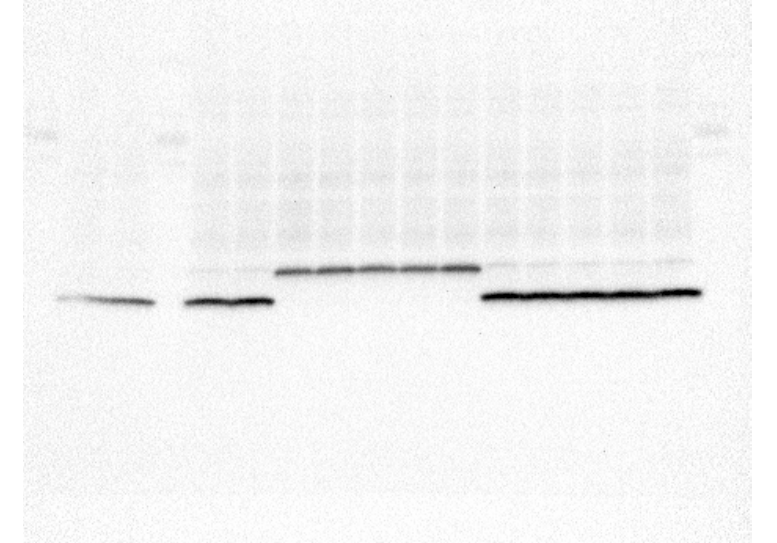

Supplement: Figure 3—figure supplement 1—source data 4. [file elife-78695-fig3-figsupp1-data4.zip › Figure 3-figure supplement 1--Source Data 4--barnalab 2019-07-26 22hr 00min_Exposure_127.2sec.tif]

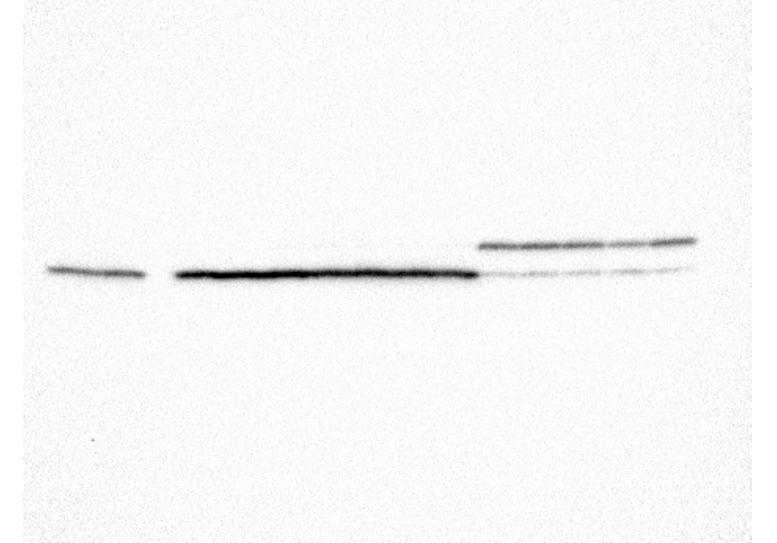

Supplement: Figure 3—figure supplement 1—source data 5. [file elife-78695-fig3-figsupp1-data5.zip › Figure 3-figure supplement 1--Source Data 5--barnalab 2019-07-26 22hr 33min-1_Exposure_3.0sec.tif]

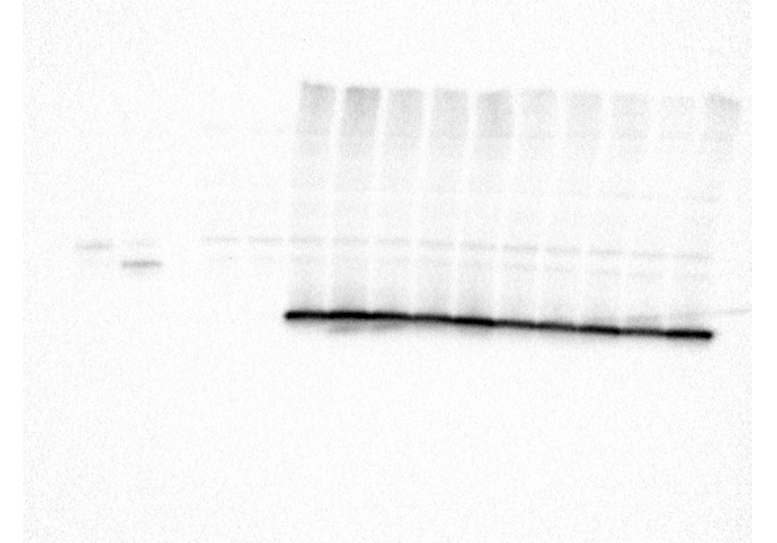

Supplement: Figure 3—figure supplement 1—source data 6. [file elife-78695-fig3-figsupp1-data6.zip › Figure 3-figure supplement 1--Source Data 6--barnalab 2019-07-26 21hr 53min_Exposure_7.2sec.tif]

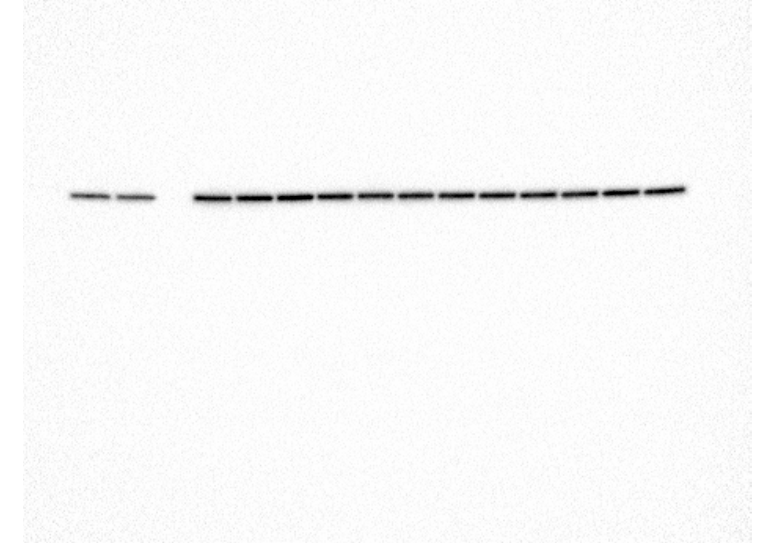

Supplement: Figure 3—figure supplement 1—source data 7. [file elife-78695-fig3-figsupp1-data7.zip › Figure 3-figure supplement 1--Source Data 7--barnalab 2019-08-07 13hr 10min_Exposure_1.3sec.tif]

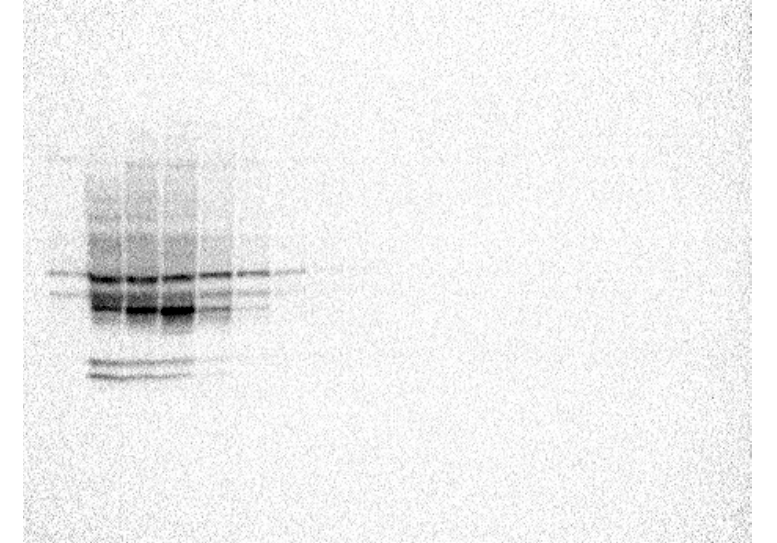

Supplement: Figure 3—figure supplement 1—source data 8. [file elife-78695-fig3-figsupp1-data8.zip › Figure 3-figure supplement 1--Source Data 8--barnalab 2019-08-11 19hr 58min_Exposure_21.3sec.tif]

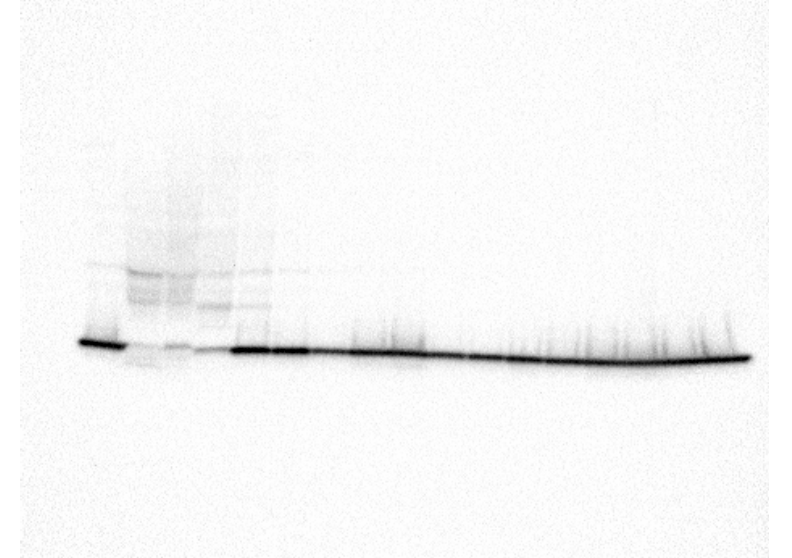

Supplement: Figure 3—figure supplement 1—source data 9. [file elife-78695-fig3-figsupp1-data9.zip › Figure 3-figure supplement 1--Source Data 9--2019.08.11.S27-3xFLAG.2C3.gradient.FLAG.WB 6.1 sec.tif]

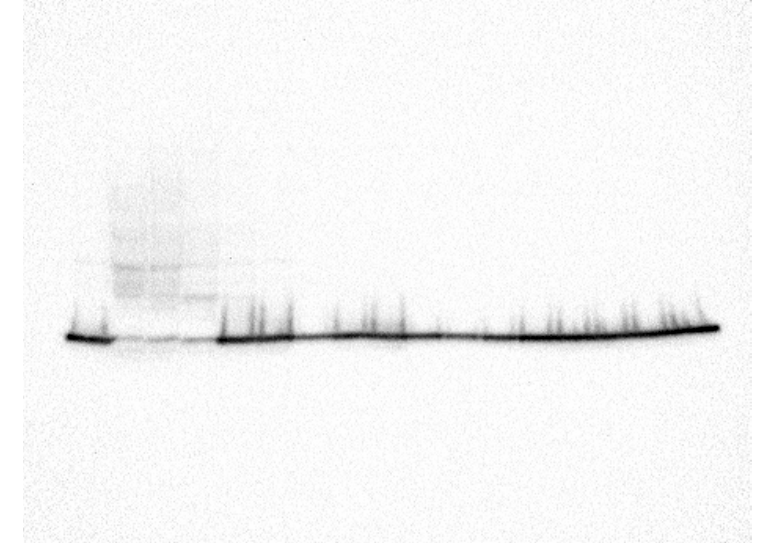

Supplement: Figure 3—figure supplement 1—source data 10. [file elife-78695-fig3-figsupp1-data10.zip › Figure 3-figure supplement 1--Source Data 10--barnalab 2019-08-11 20hr 15min_Exposure_6.1sec.tif]

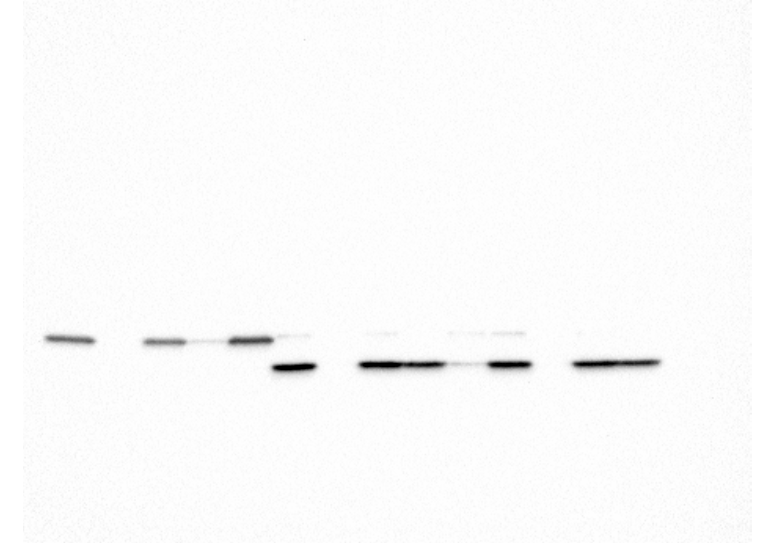

Supplement: Figure 3—figure supplement 1—source data 11. [file elife-78695-fig3-figsupp1-data11.zip › Figure 3-figure supplement 1--Source Data 11--barnalab 2019-10-14 16hr 30min_Exposure_11.2sec.tif]

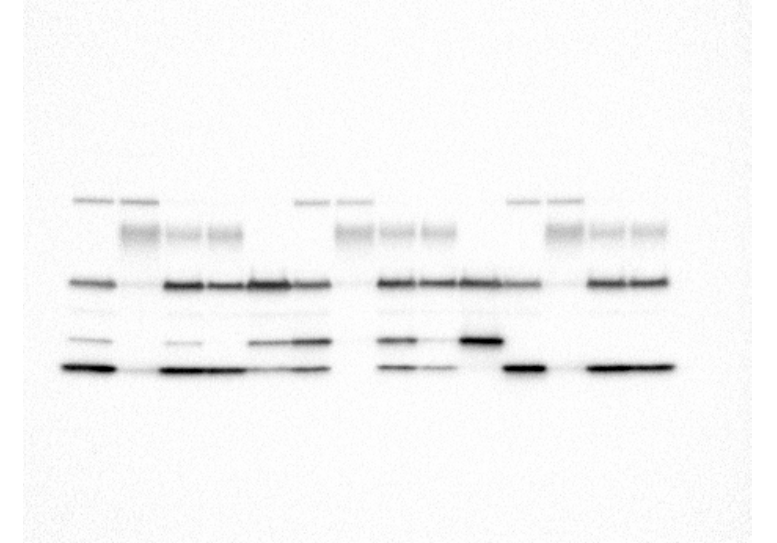

Supplement: Figure 3—figure supplement 1—source data 12. [file elife-78695-fig3-figsupp1-data12.zip › Figure 3-figure supplement 1--Source Data 12--barnalab 2019-10-18 20hr 31min_Exposure_5.0sec.tif]

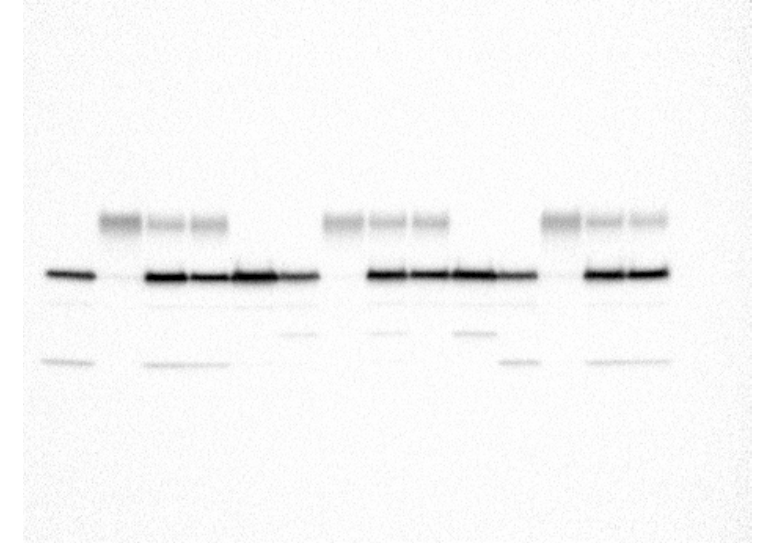

Supplement: Figure 3—figure supplement 1—source data 13. [file elife-78695-fig3-figsupp1-data13.zip › Figure 3-figure supplement 1--Source Data 13--barnalab 2019-10-16 19hr 03min_Exposure_8.0sec.tif]

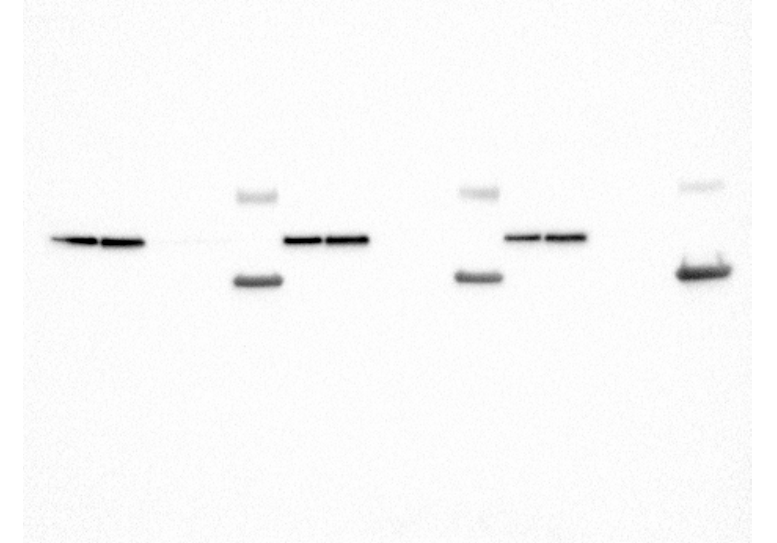

Supplement: Figure 3—figure supplement 1—source data 14. [file elife-78695-fig3-figsupp1-data14.zip › Figure 3-figure supplement 1--Source Data 14--barnalab 2019-10-23 17hr 33min_Exposure_21.6sec.tif]

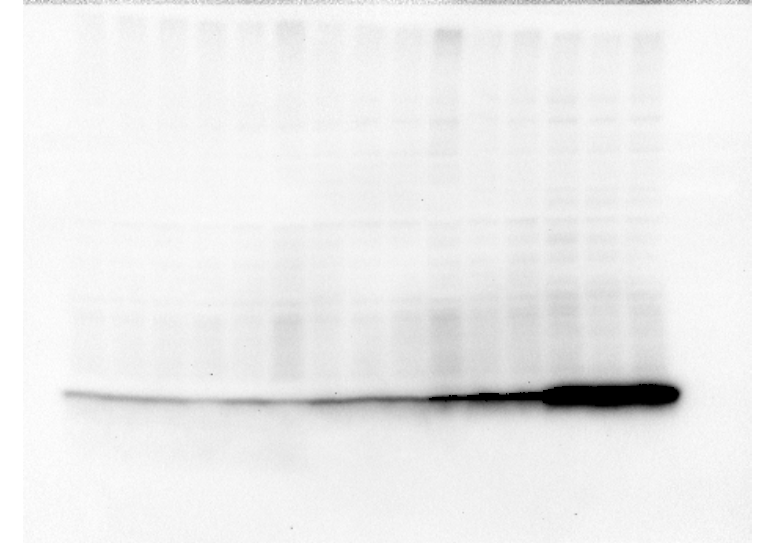

Supplement: Figure 4—source data 1. [file elife-78695-fig4-data1.zip › Figure 4--Source Data 1--Barna Lab 2022-10-06_23h27m36s_Exposure_112.7sec.tif]

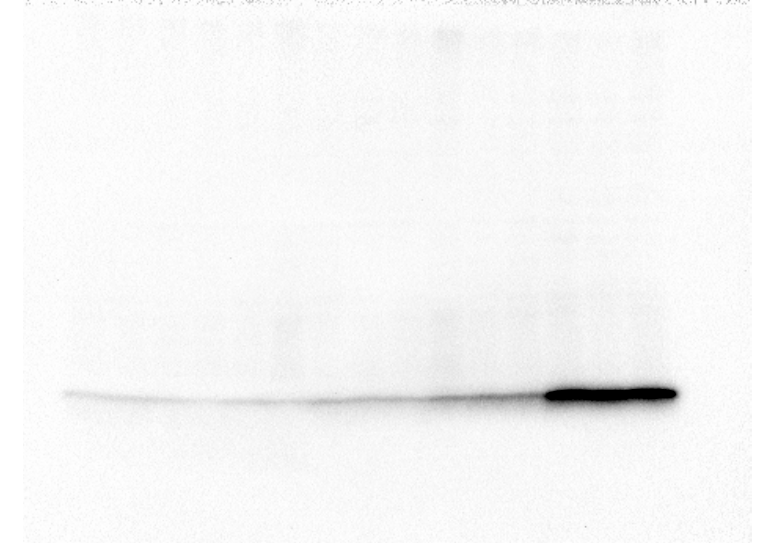

Supplement: Figure 4—source data 2. [file elife-78695-fig4-data2.zip › Figure 4--Source Data 2--Barna Lab 2022-10-06_23h27m36s_Exposure_11.2sec.tif]

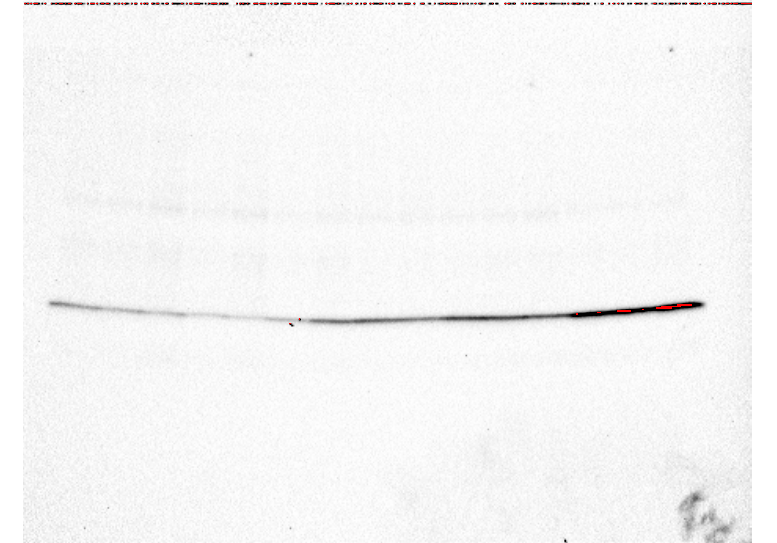

Supplement: Figure 4—source data 3. [file elife-78695-fig4-data3.zip › Figure 4--Source Data 3--Barna Lab 2022-11-03_15h42m54s_Exposure_300.0sec.tif]

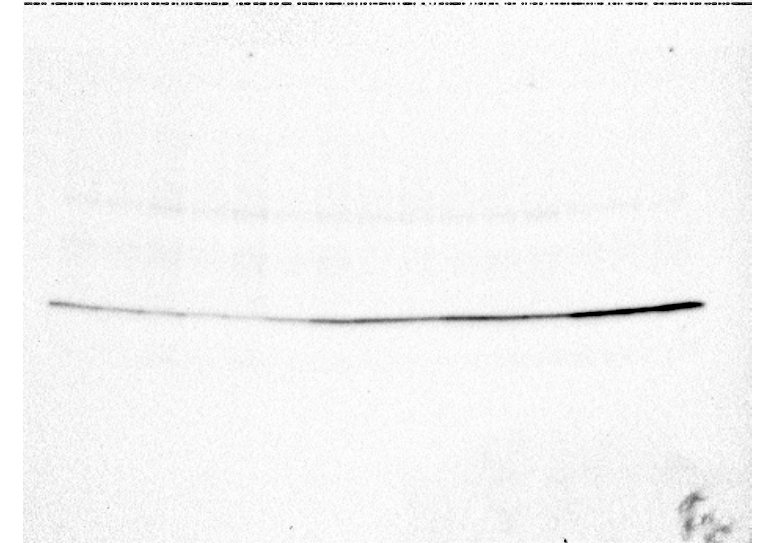

Supplement: Figure 4—source data 4. [file elife-78695-fig4-data4.zip › Figure 4--Source Data 4--Barna Lab 2022-11-03_15h42m54s_Exposure_227.8sec.tif]

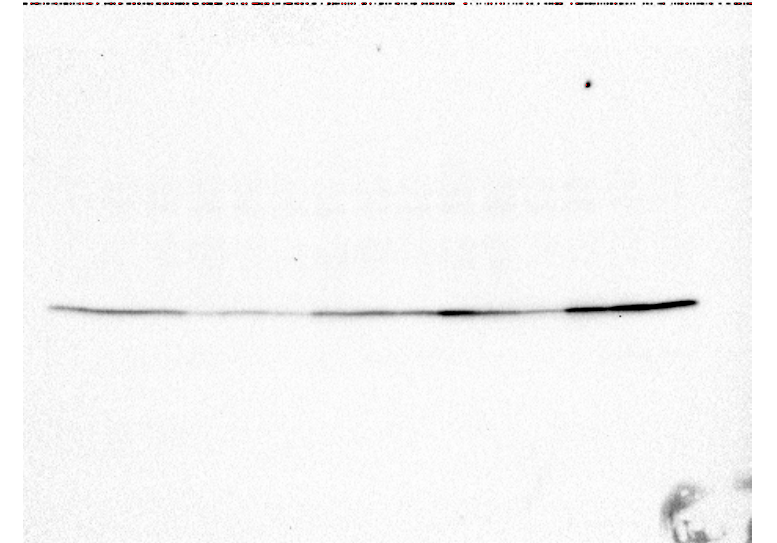

Supplement: Figure 4—source data 5. [file elife-78695-fig4-data5.zip › Figure 4--Source Data 5--Barna Lab 2022-11-03_15h33m09s_Exposure_166.0sec.tif]

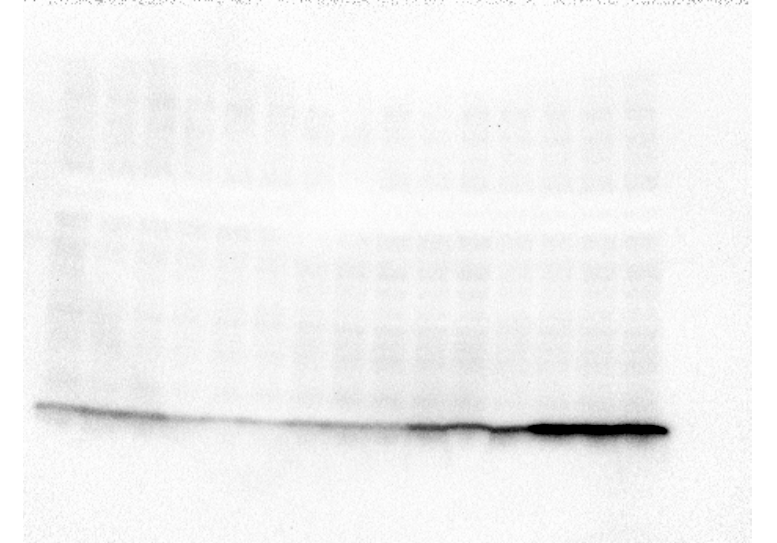

Supplement: Figure 4—source data 6. [file elife-78695-fig4-data6.zip › Figure 4--Source Data 6--Barna Lab 2022-10-06_23h20m33s_Exposure_11.2sec.tif]

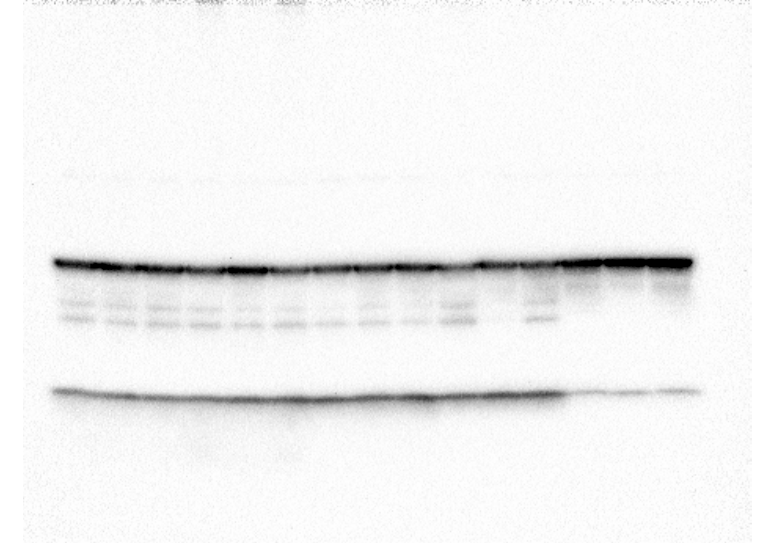

Supplement: Figure 4—source data 7. [file elife-78695-fig4-data7.zip › Figure 4--Source Data 7--Barna Lab 2022-10-08_20h49m06s_Exposure_11.1sec.tif]

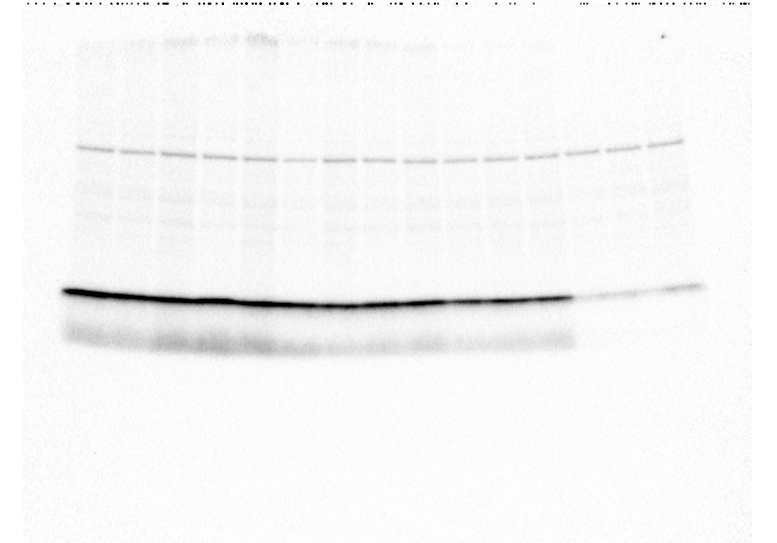

Supplement: Figure 4—source data 8. [file elife-78695-fig4-data8.zip › Figure 4--Source Data 8--Barna Lab 2022-10-06_22h52m48s_Exposure_11.1sec.tif]

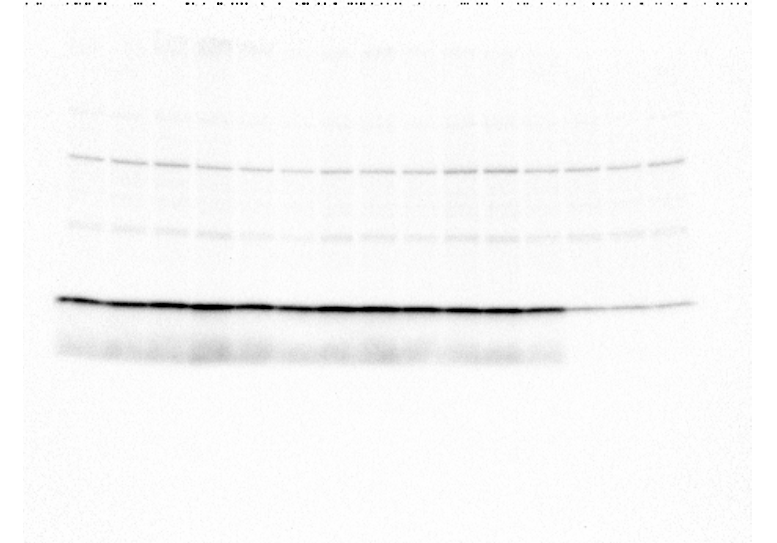

Supplement: Figure 4—source data 9. [file elife-78695-fig4-data9.zip › Figure 4--Source Data 9--Barna Lab 2022-10-06_22h45m42s_Exposure_11.1sec.tif]

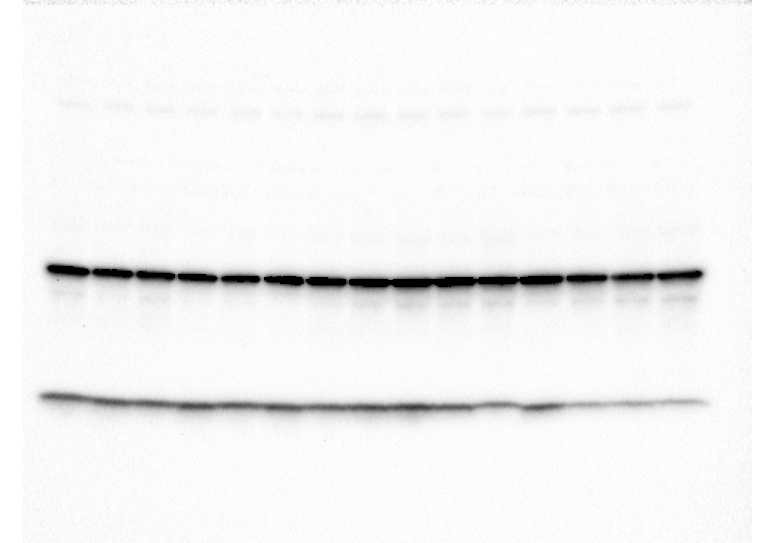

Supplement: Figure 4—source data 10. [file elife-78695-fig4-data10.zip › Figure 4--Source Data 10--Barna Lab 2022-10-08_20h58m53s_Exposure_92.2sec.tif]

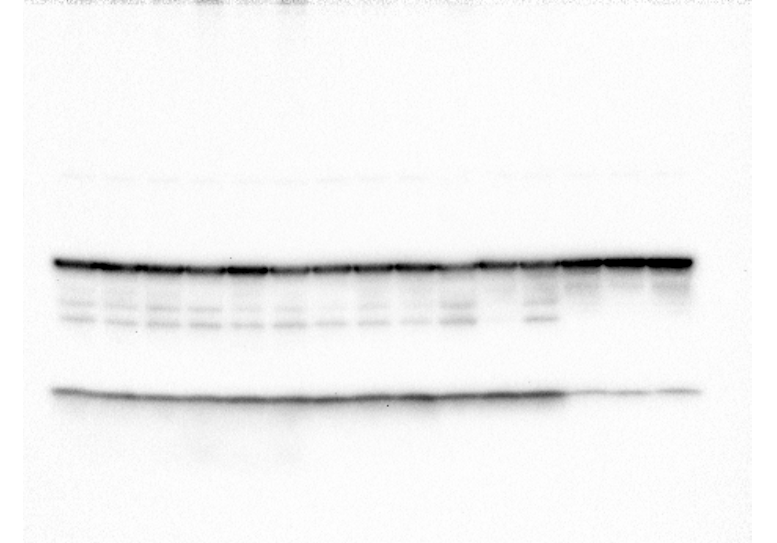

Supplement: Figure 4—source data 11. [file elife-78695-fig4-data11.zip › Figure 4--Source Data 11--Barna Lab 2022-10-08_20h49m06s_Exposure_26.3sec.tif]

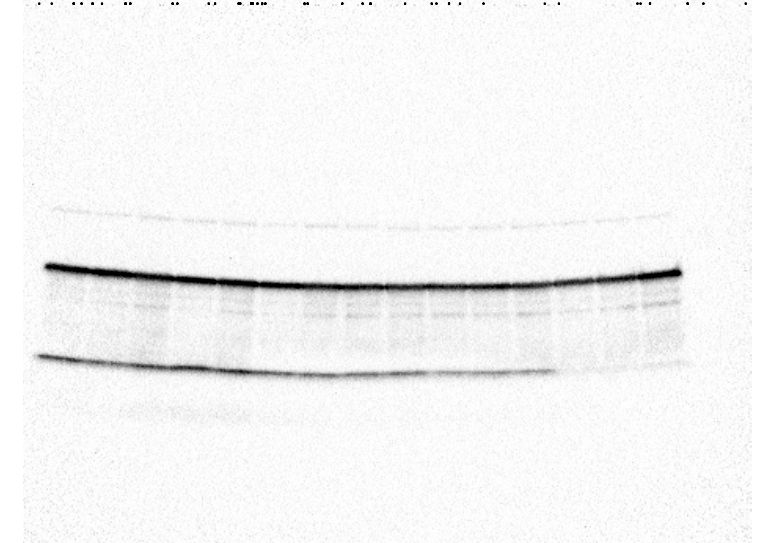

Supplement: Figure 4—source data 12. [file elife-78695-fig4-data12.zip › Figure 4--Source Data 12--Barna Lab 2022-10-08_21h16m47s_Exposure_6.1sec.tif]

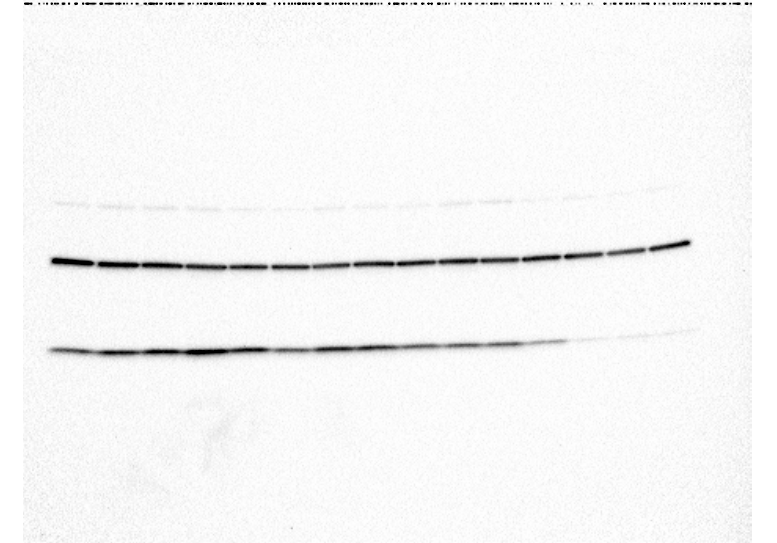

Supplement: Figure 4—source data 13. [file elife-78695-fig4-data13.zip › Figure 4--Source Data 13--Barna Lab 2022-10-08_21h07m18s_Exposure_66.9sec.tif]

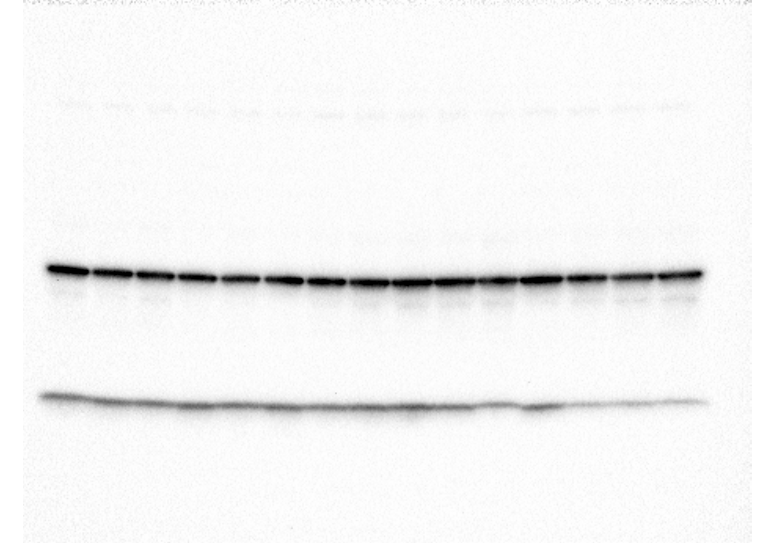

Supplement: Figure 4—source data 13. [file elife-78695-fig4-data13.zip › Figure 4--Source Data 14--Barna Lab 2022-10-08_20h58m53s_Exposure_31.4sec.tif]

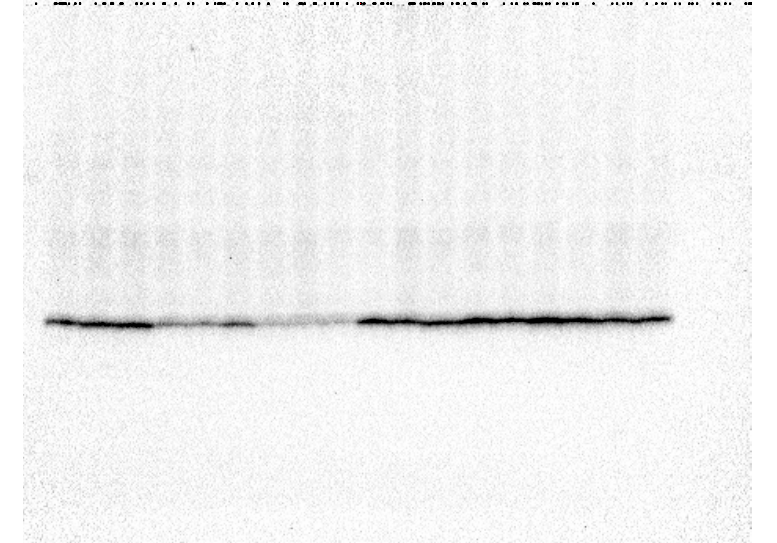

Supplement: Figure 5—source data 1. [file elife-78695-fig5-data1.zip › Figure 5--Source Data 1--Barna Lab 2022-11-03_14h47m39s_Exposure_11.1sec.tif]

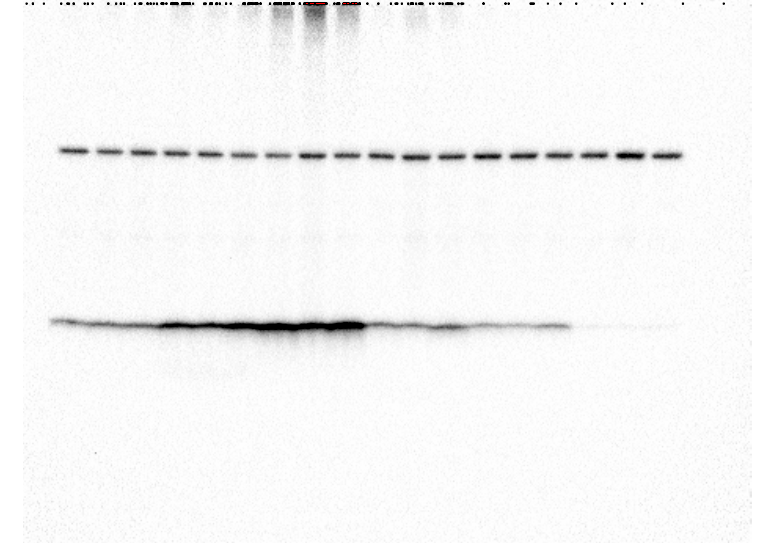

Supplement: Figure 5—source data 2. [file elife-78695-fig5-data2.zip › Figure 5--Source Data 2--Barna Lab 2022-11-04_14h20m35s_Exposure_4.8sec.tif]

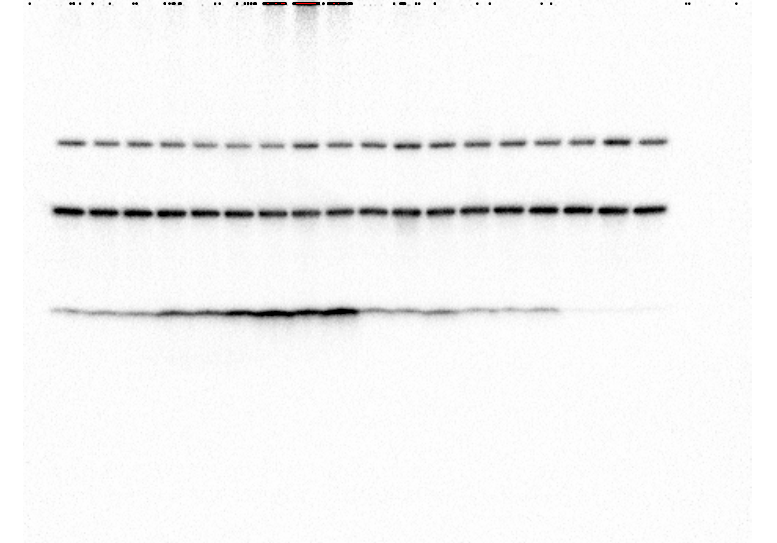

Supplement: Figure 5—source data 3. [file elife-78695-fig5-data3.zip › Figure 5--Source Data 3--Barna Lab 2022-11-05_19h27m46s_Exposure_1.0sec.tif]

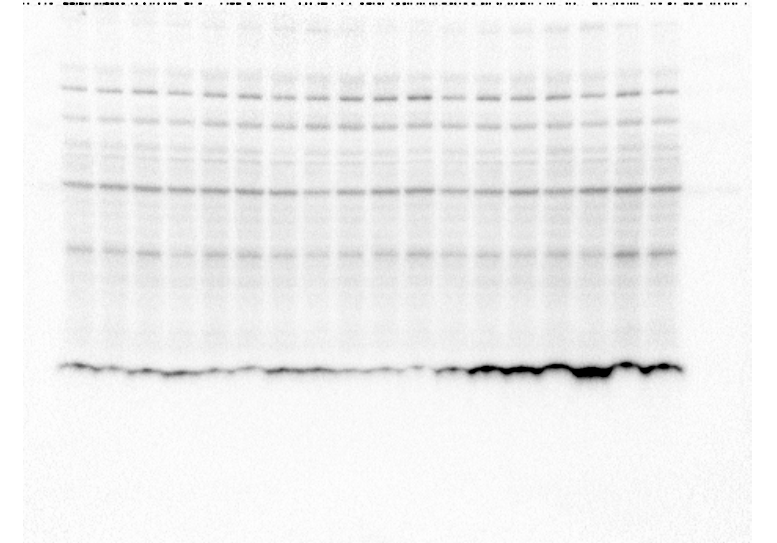

Supplement: Figure 5—source data 4. [file elife-78695-fig5-data4.zip › Figure 5--Source Data 4--Barna Lab 2022-11-05_19h12m00s_Exposure_6.1sec.tif]

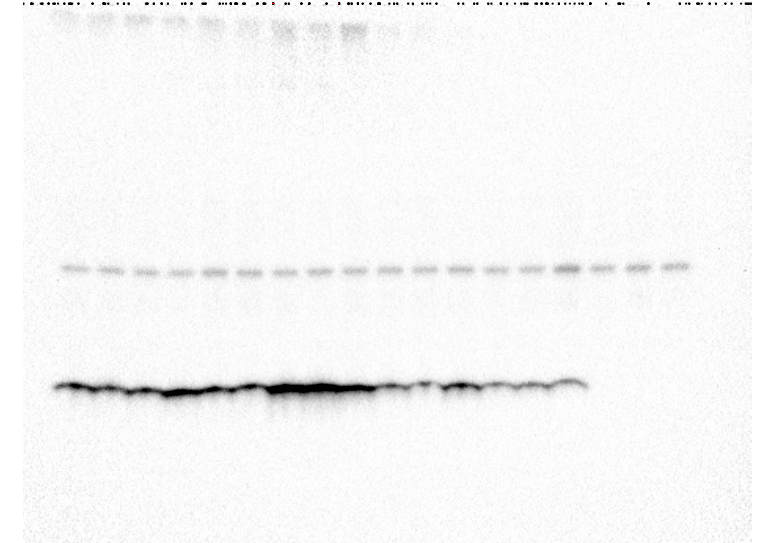

Supplement: Figure 5—source data 5. [file elife-78695-fig5-data5.zip › Figure 5--Source Data 5--Barna Lab 2022-11-06_11h31m11s_Exposure_16.2sec.tif]

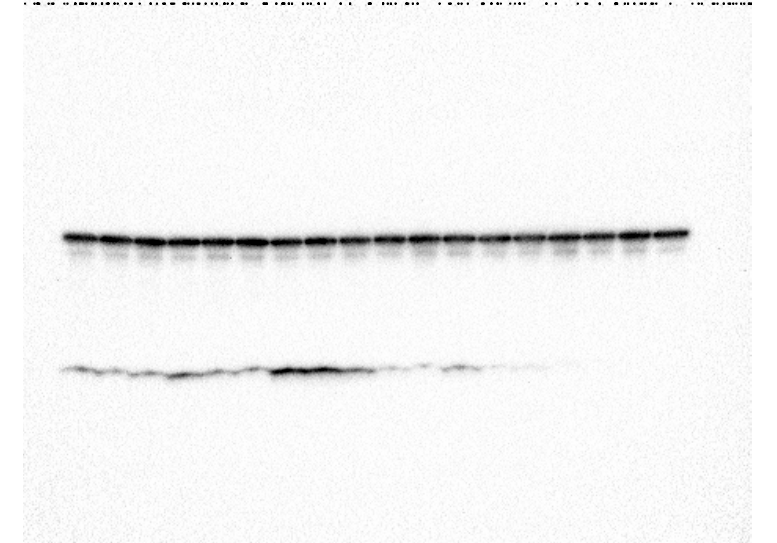

Supplement: Figure 5—source data 6. [file elife-78695-fig5-data6.zip › Figure 5--Source Data 6--Barna Lab 2022-11-07_16h33m53s_Exposure_13.3sec.tif]

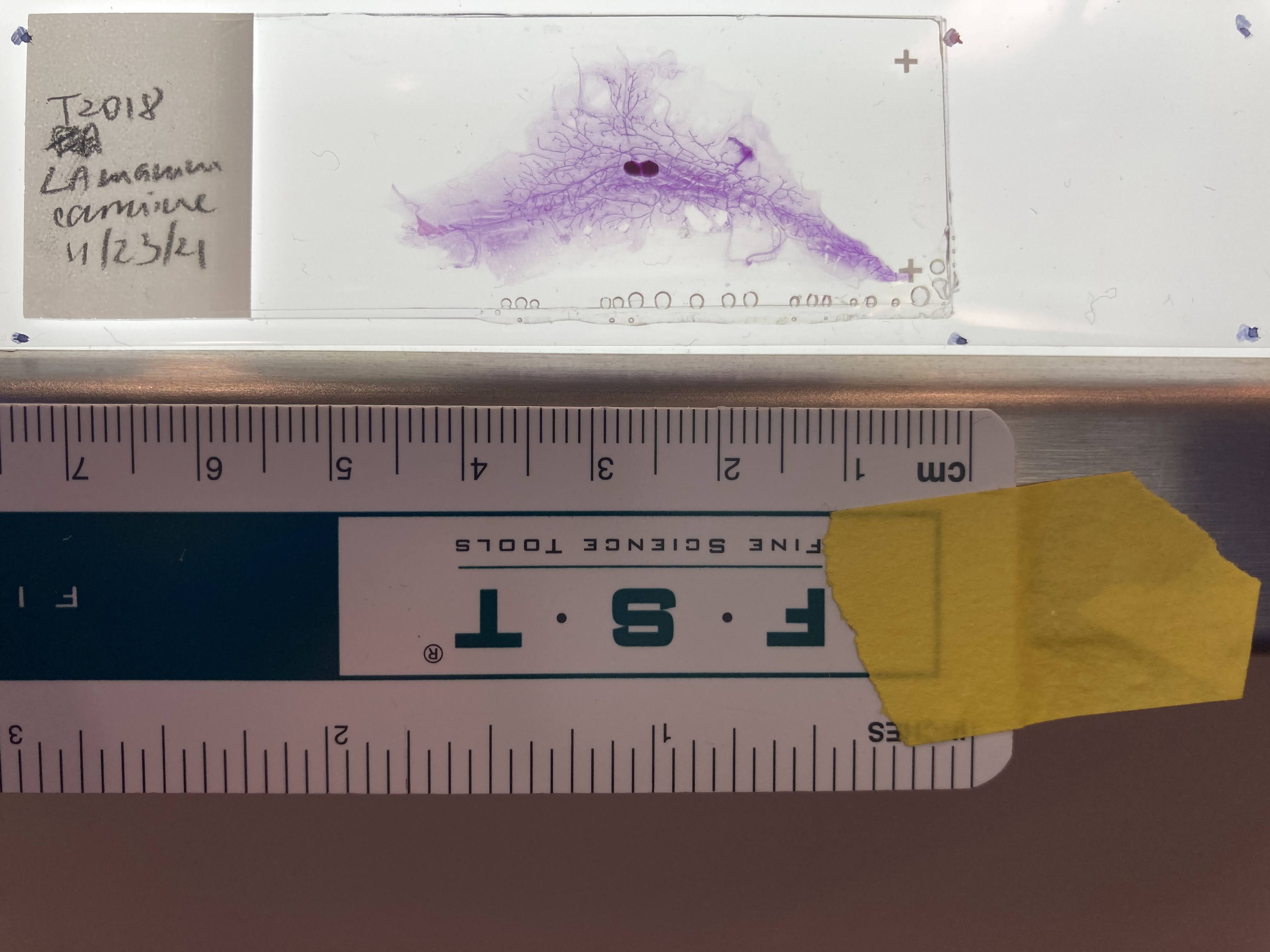

Supplement: Figure 6—source data 1. [file elife-78695-fig6-data1.zip › Figure 6--Source Data 1--T2018.jpg]

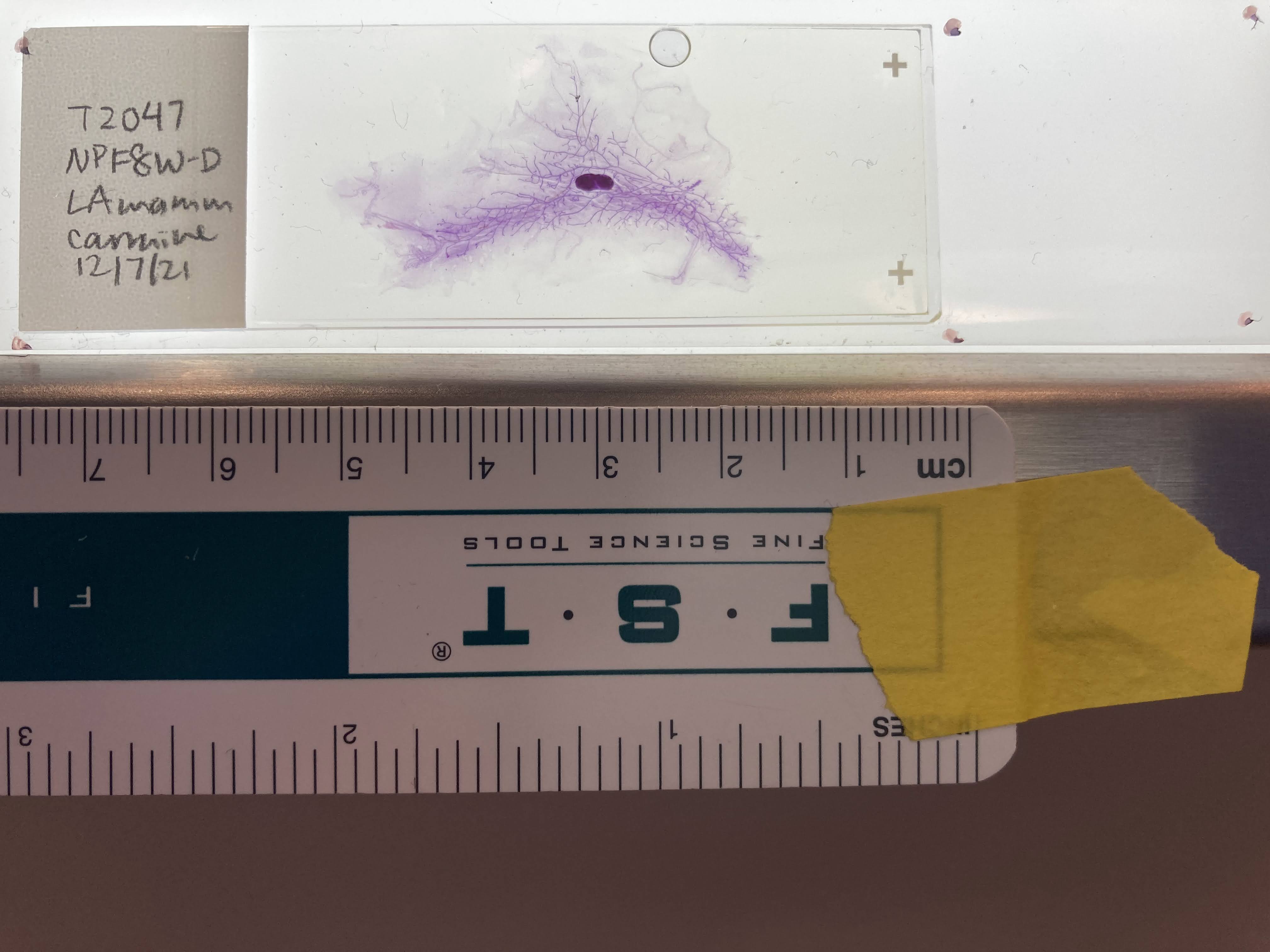

Supplement: Figure 6—source data 2. [file elife-78695-fig6-data2.zip › Figure 6--Source Data 2--T2047.jpg]

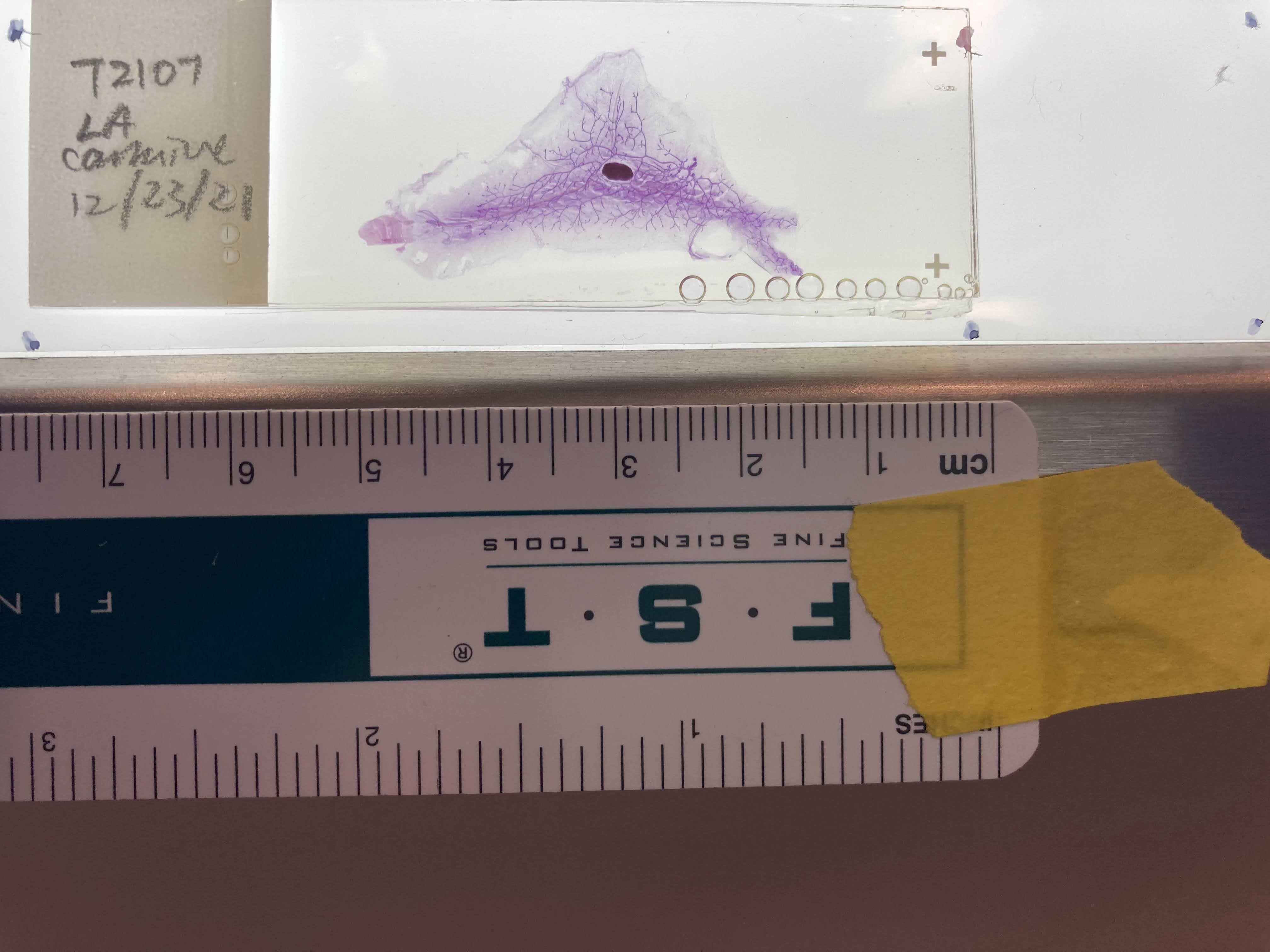

Supplement: Figure 6—source data 3. [file elife-78695-fig6-data3.zip › Figure 6--Source Data 3--T2107.jpg]

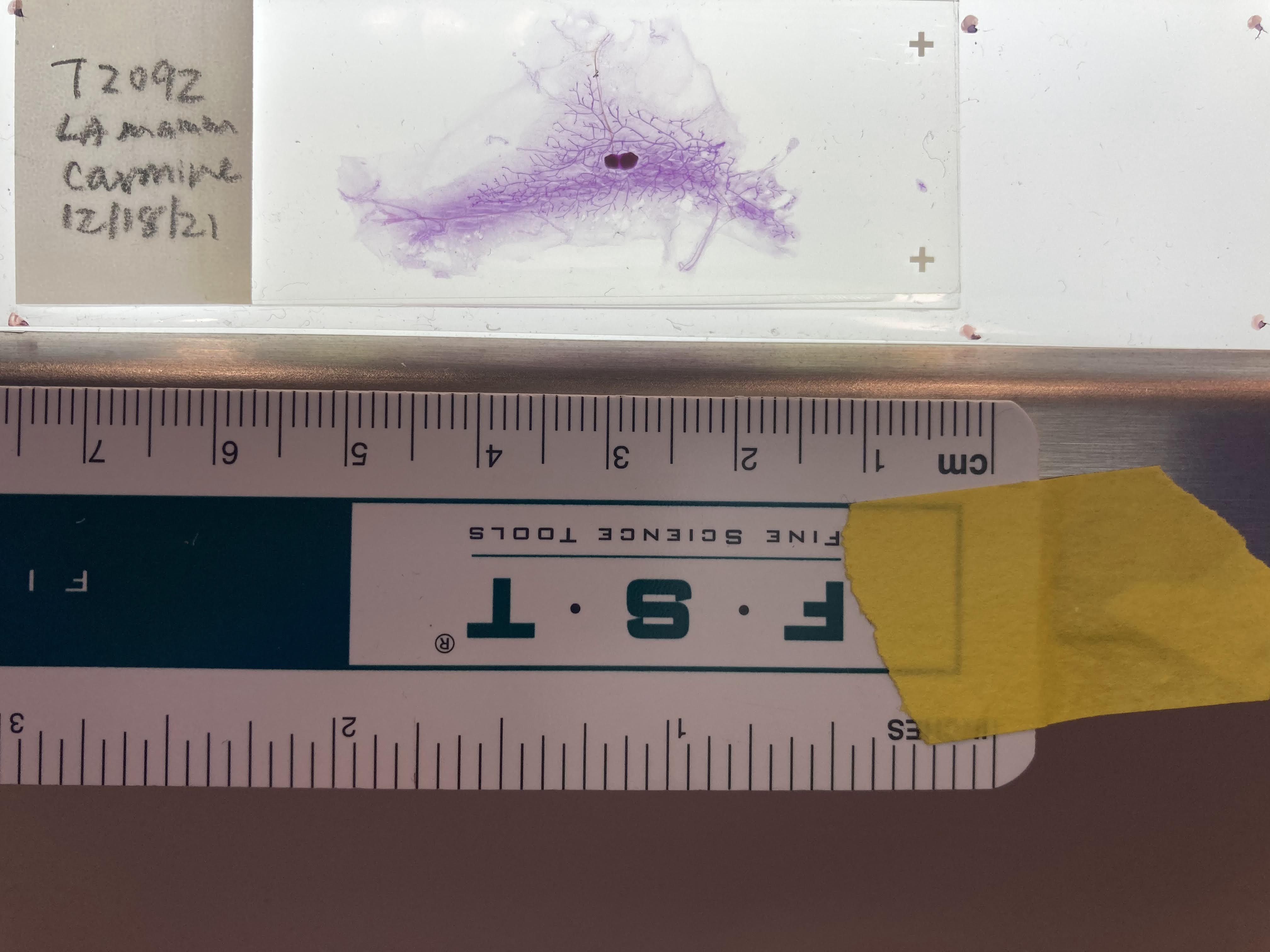

Supplement: Figure 6—source data 4. [file elife-78695-fig6-data4.zip › Figure 6--Source Data 4--T2092.jpg]

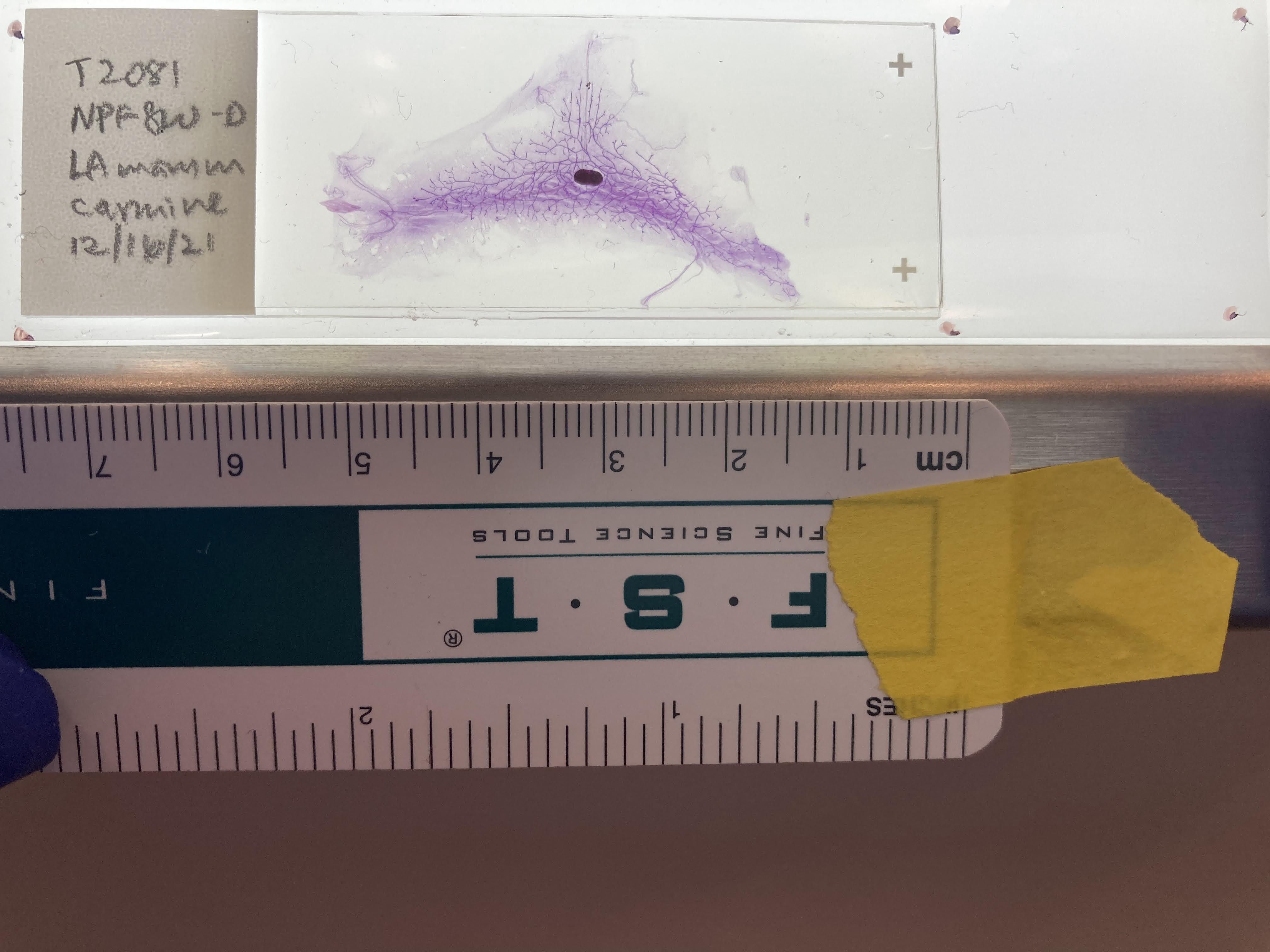

Supplement: Figure 6—source data 5. [file elife-78695-fig6-data5.zip › Figure 6--Source Data 5--T2081.jpg]

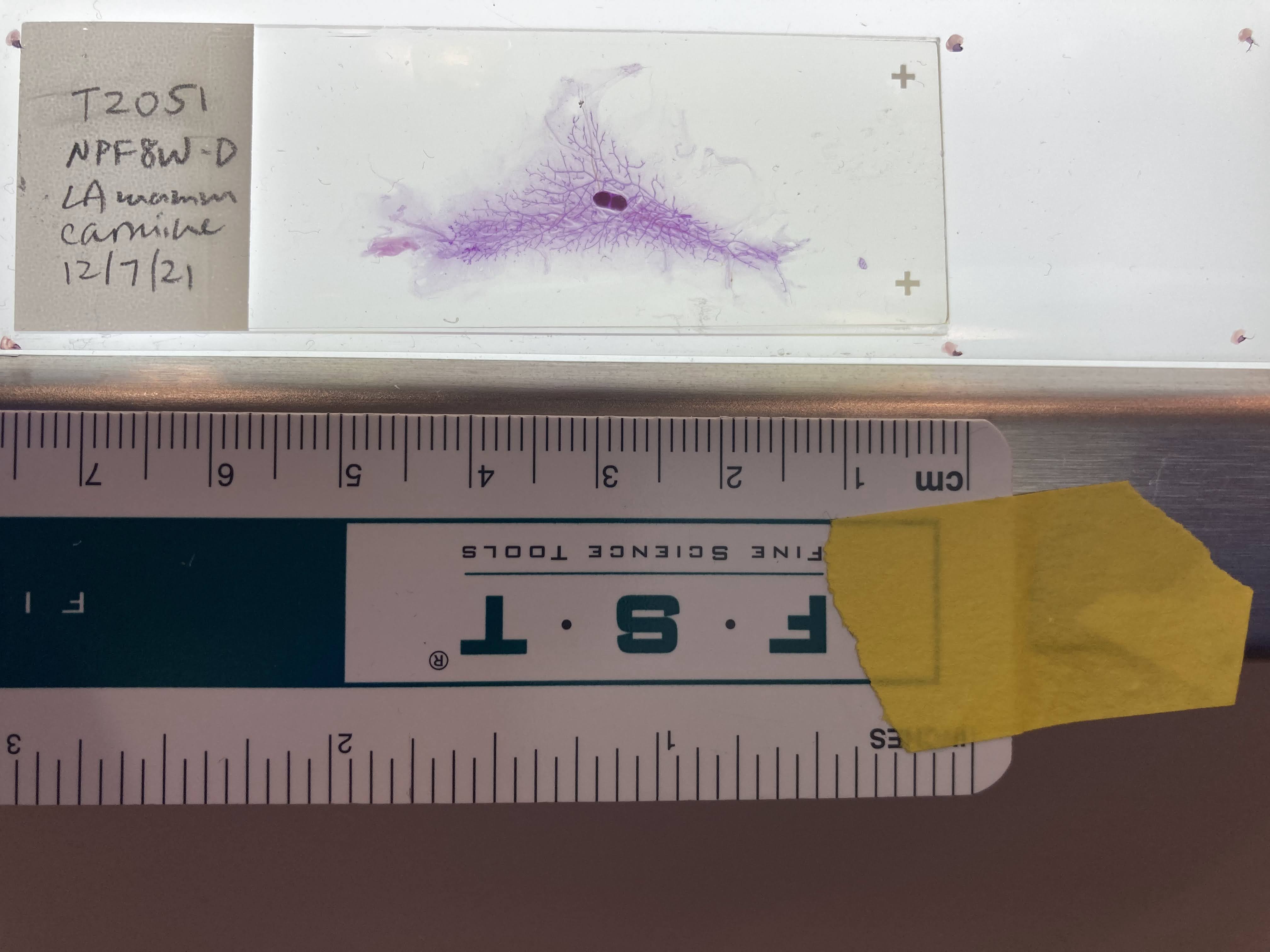

Supplement: Figure 6—source data 6. [file elife-78695-fig6-data6.zip › Figure 6--Source Data 6--T2051.jpg]

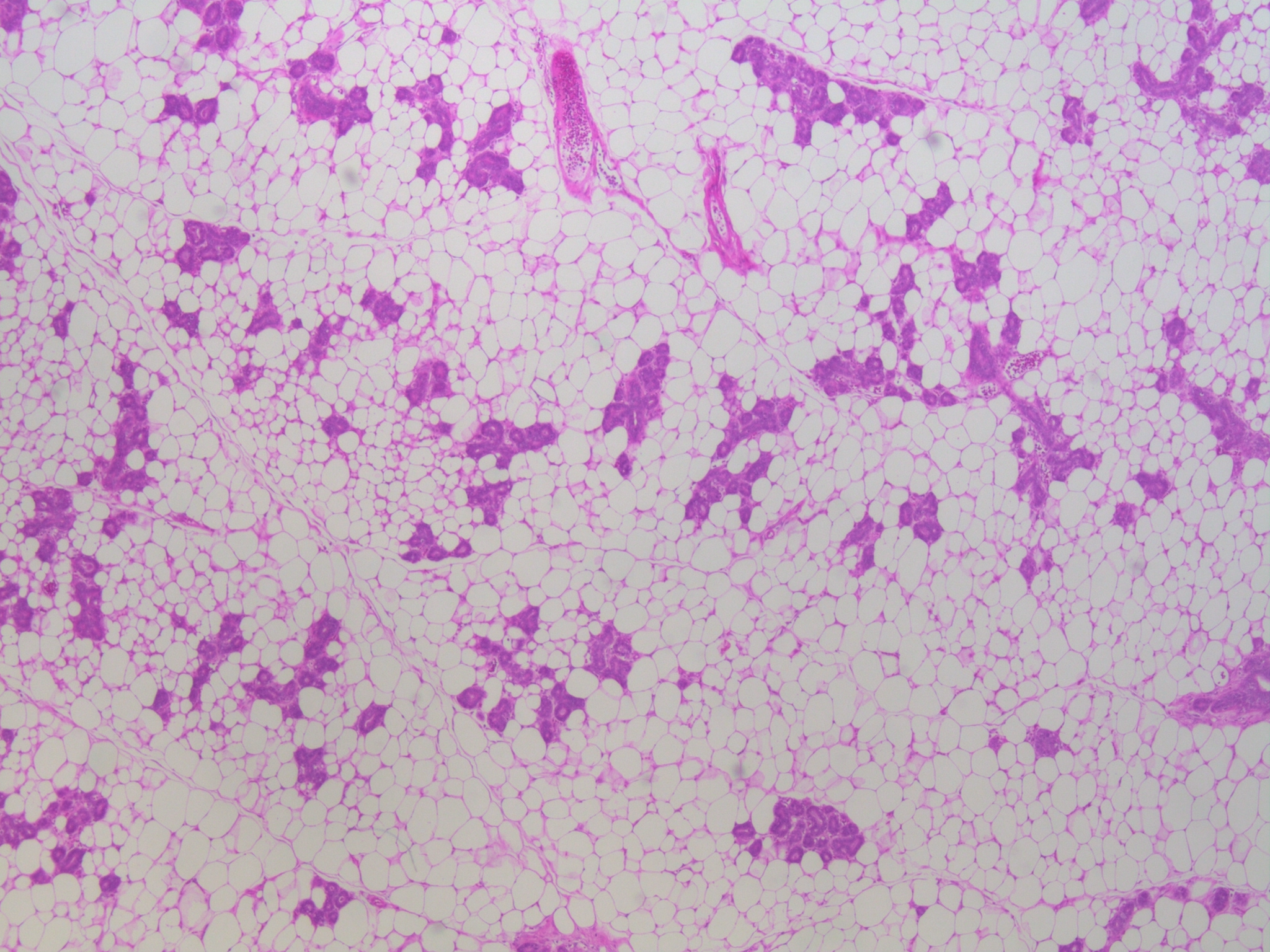

Supplement: Figure 6—source data 7. [file elife-78695-fig6-data7.zip › Figure 6--Source Data 7--5x_t1850_1.tif]

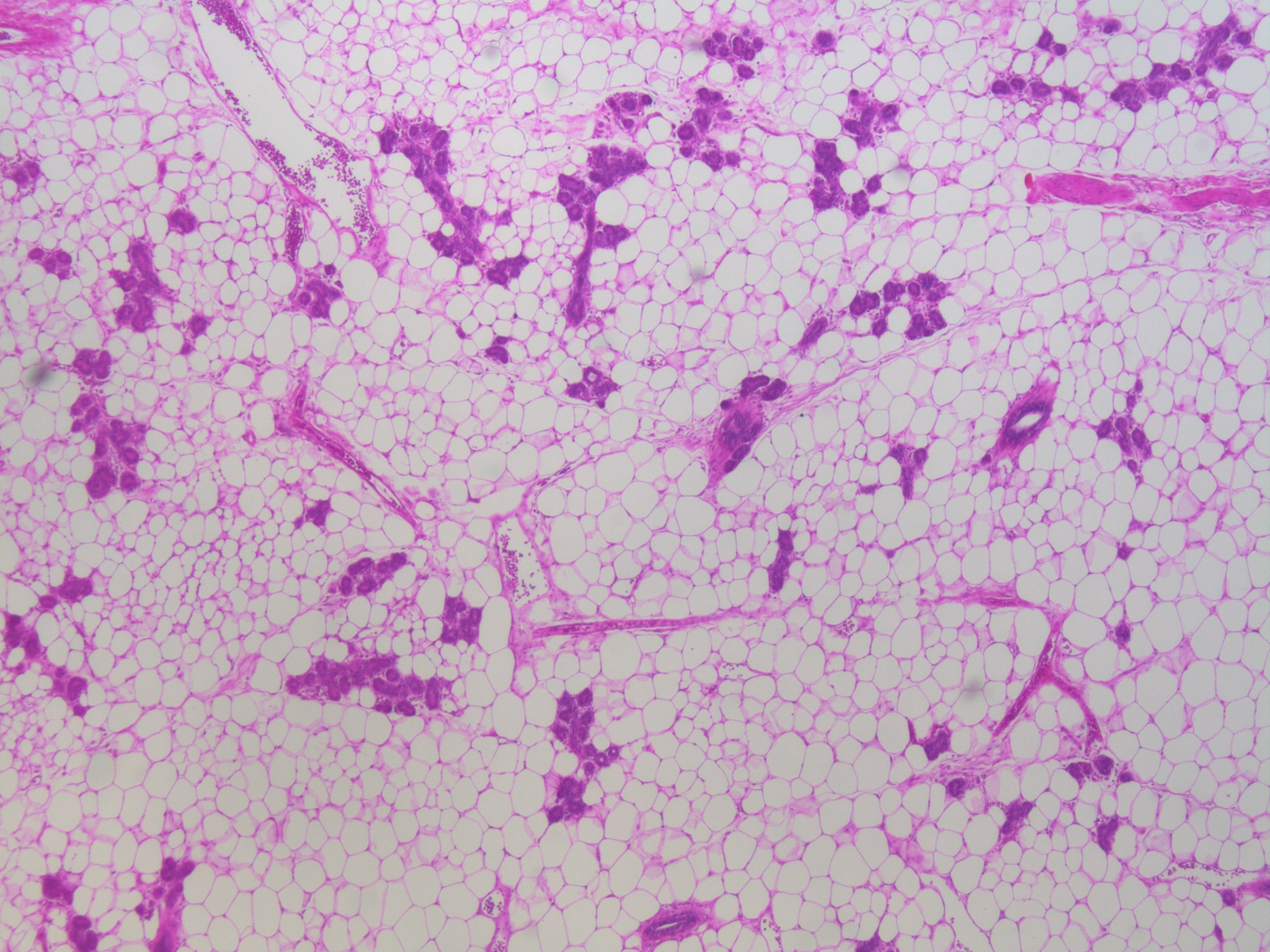

Supplement: Figure 6—source data 8. [file elife-78695-fig6-data8.zip › Figure 6--Source Data 8--5x_t1866_1.tif]

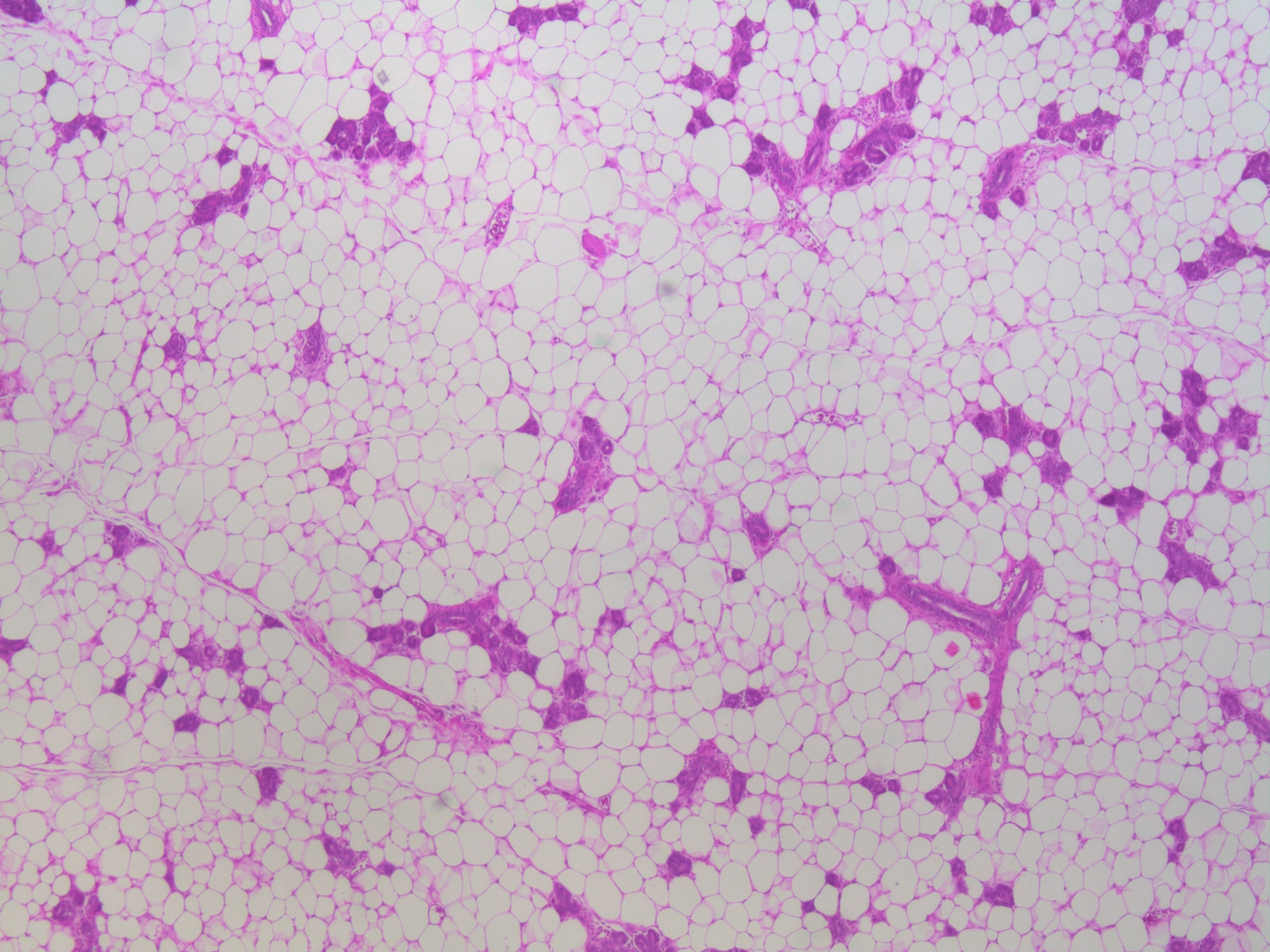

Supplement: Figure 6—source data 9. [file elife-78695-fig6-data9.zip › Figure 6--Source Data 9--5x_t1931_1.tif]

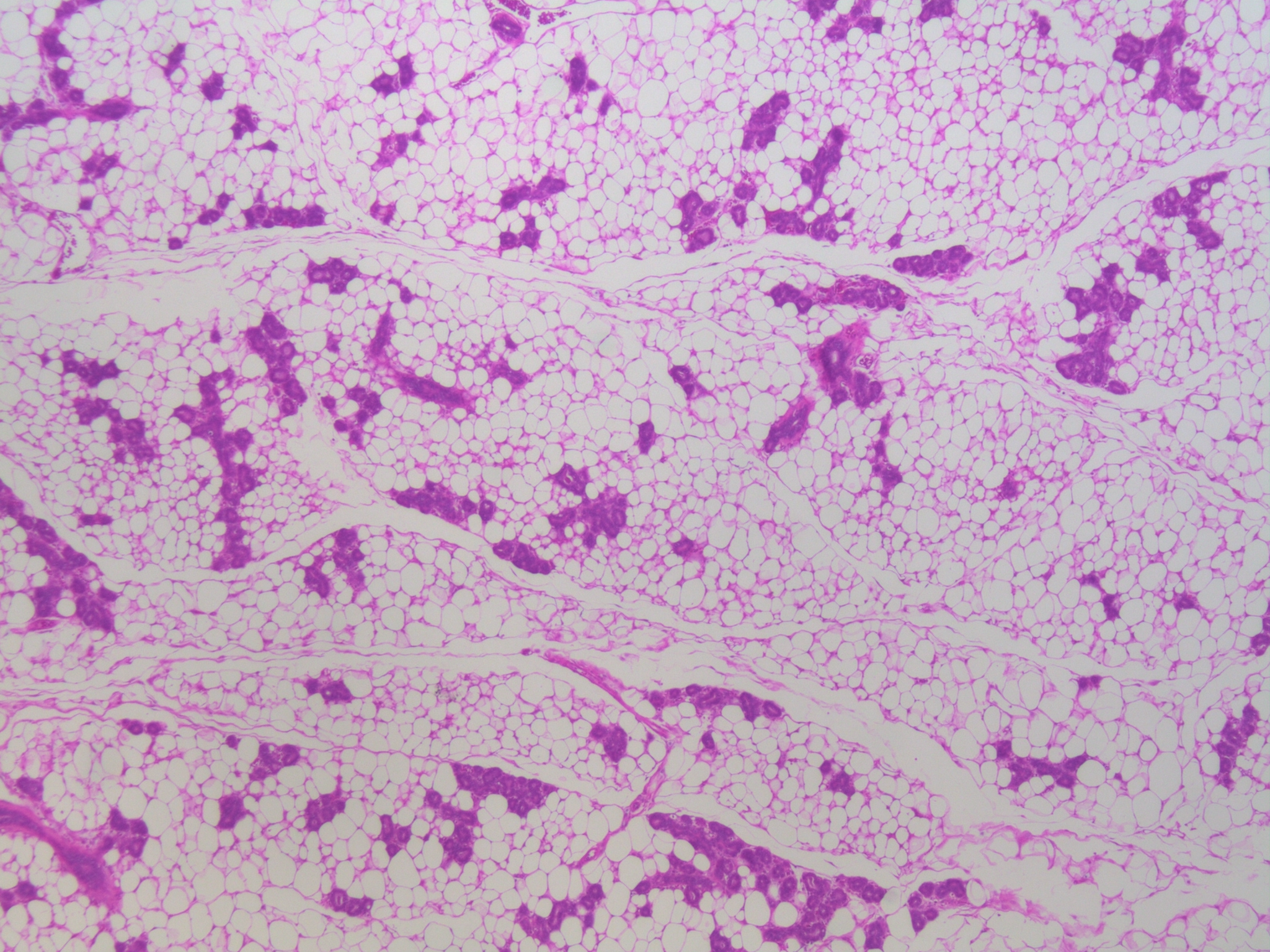

Supplement: Figure 6—source data 10. [file elife-78695-fig6-data10.zip › Figure 6--Source Data 10--5x_t1909_.tif]

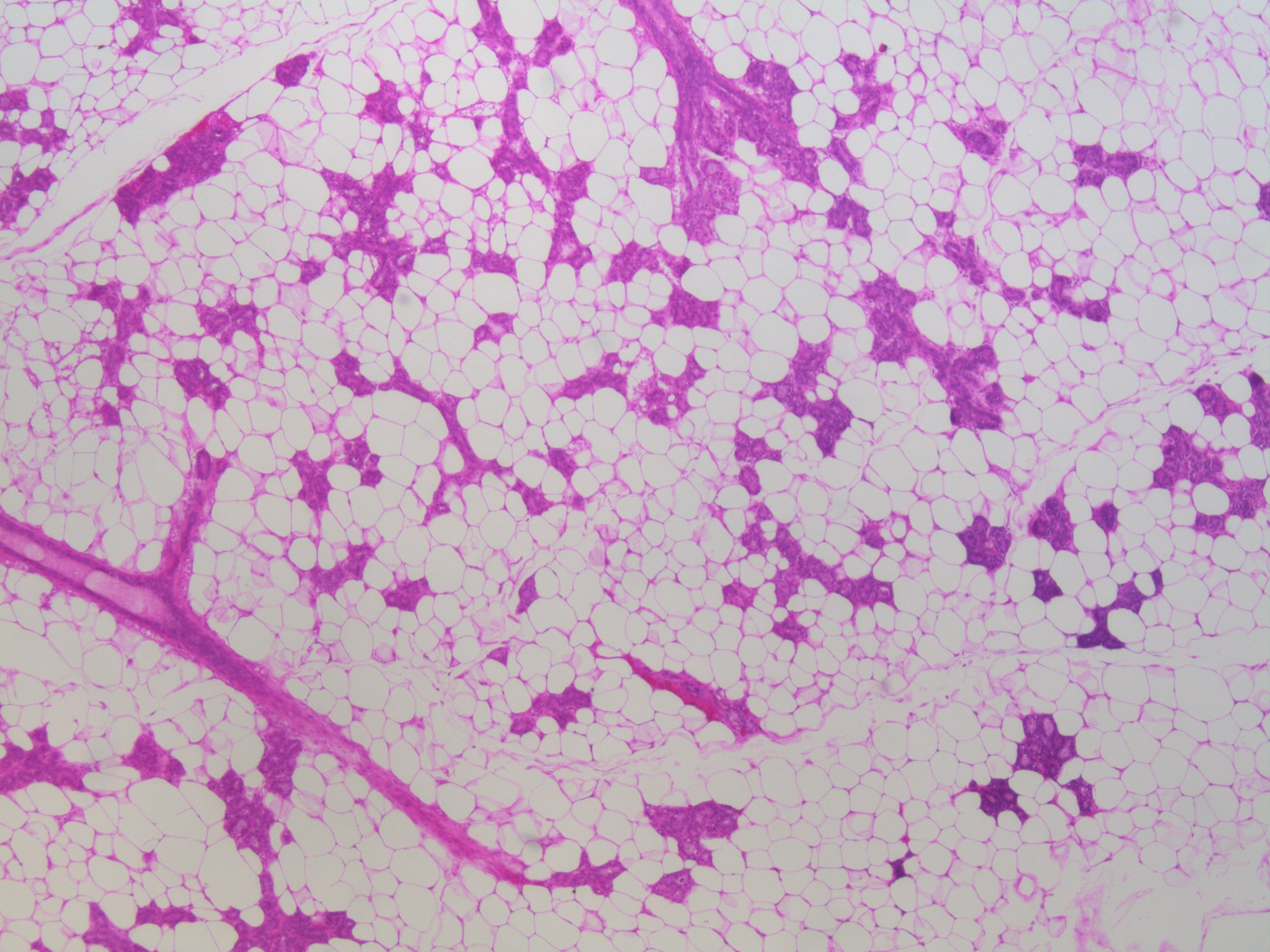

Supplement: Figure 6—source data 11. [file elife-78695-fig6-data11.zip › Figure 6--Source Data 11--5x_t1859_1.tif]

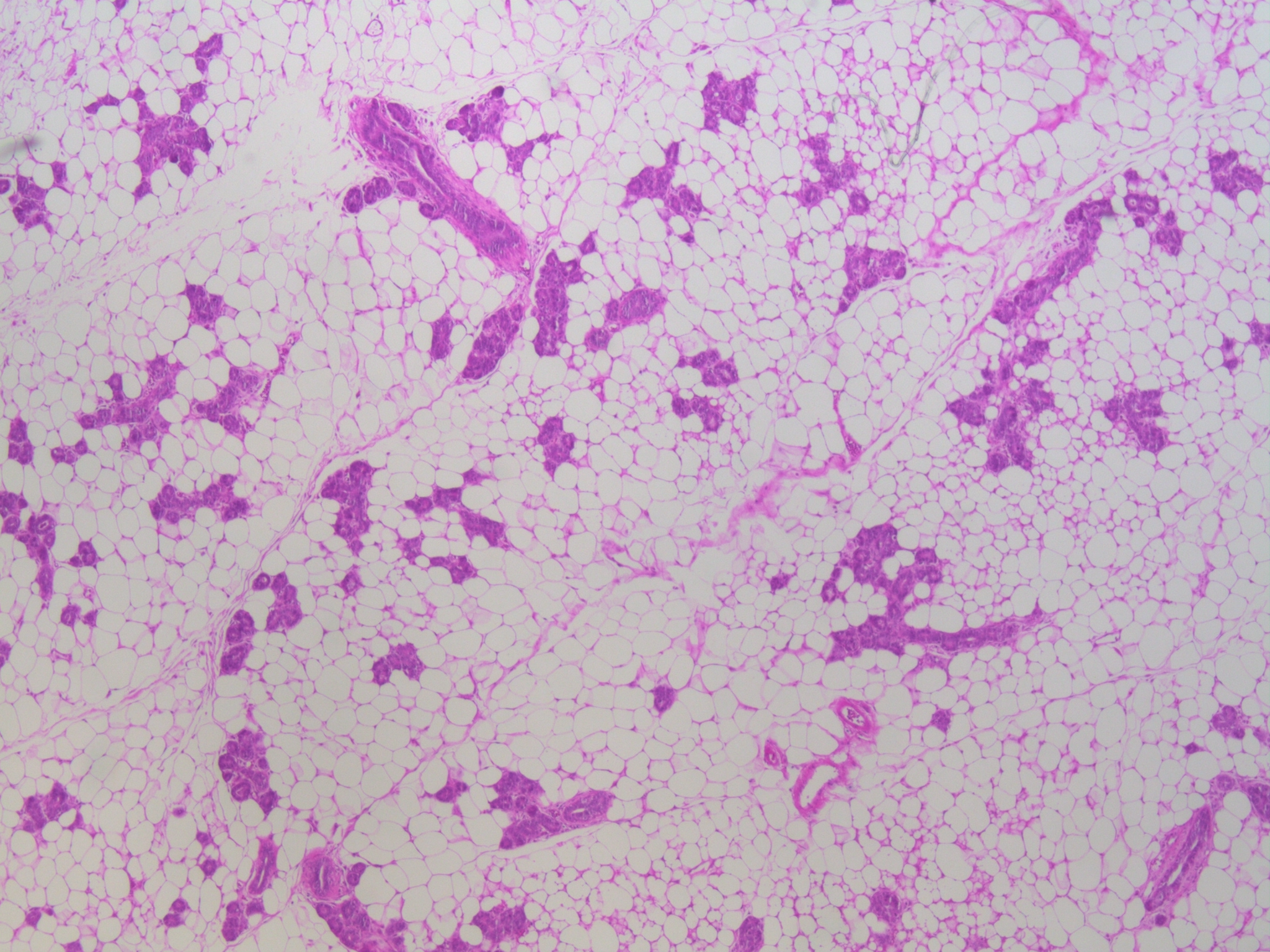

Supplement: Figure 6—source data 12. [file elife-78695-fig6-data12.zip › Figure 6--Source Data 12--5x_t1892_1.tif]

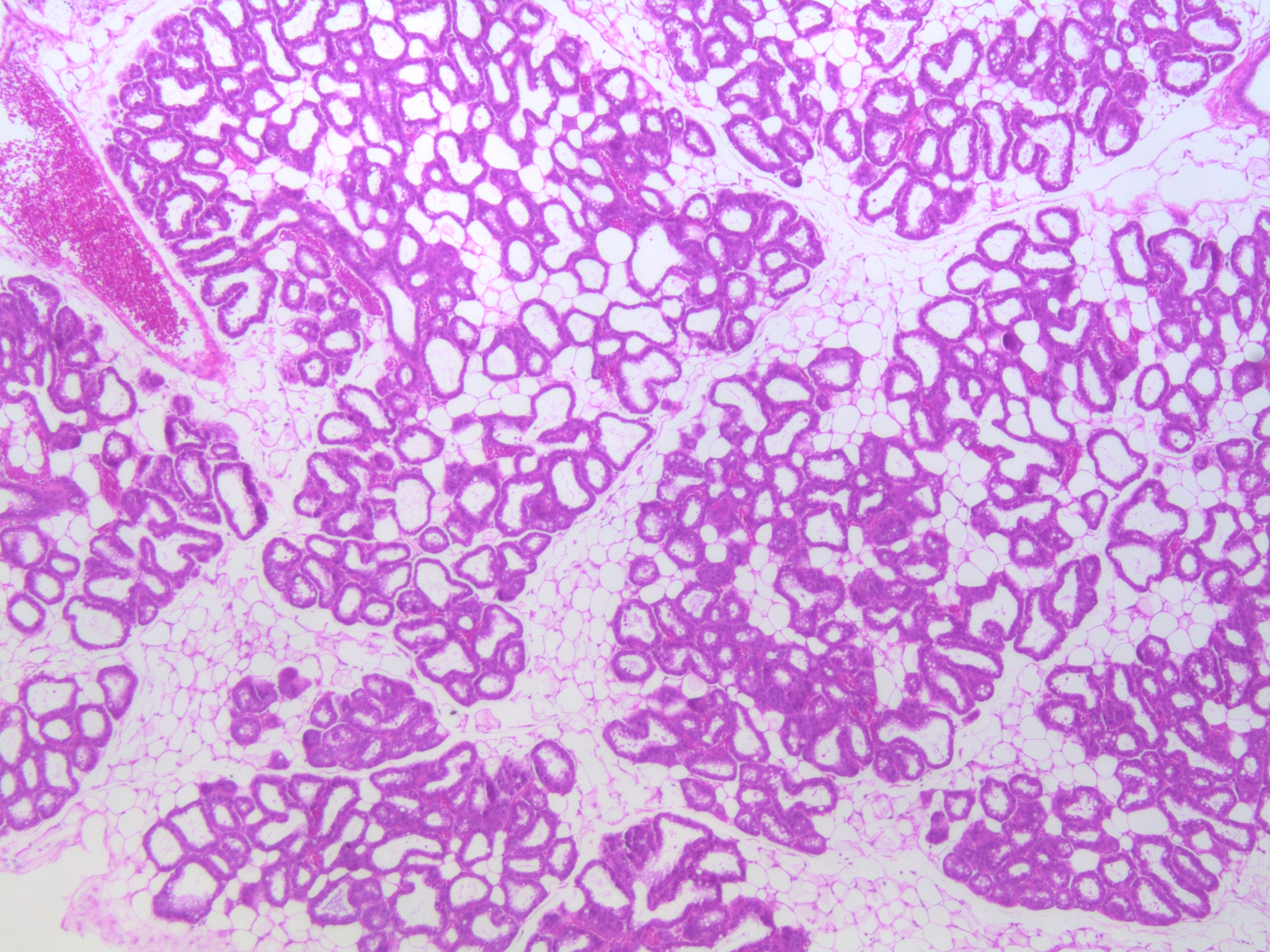

Supplement: Figure 6—source data 13. [file elife-78695-fig6-data13.zip › Figure 6--Source Data 13--5x_t915_02.tif]

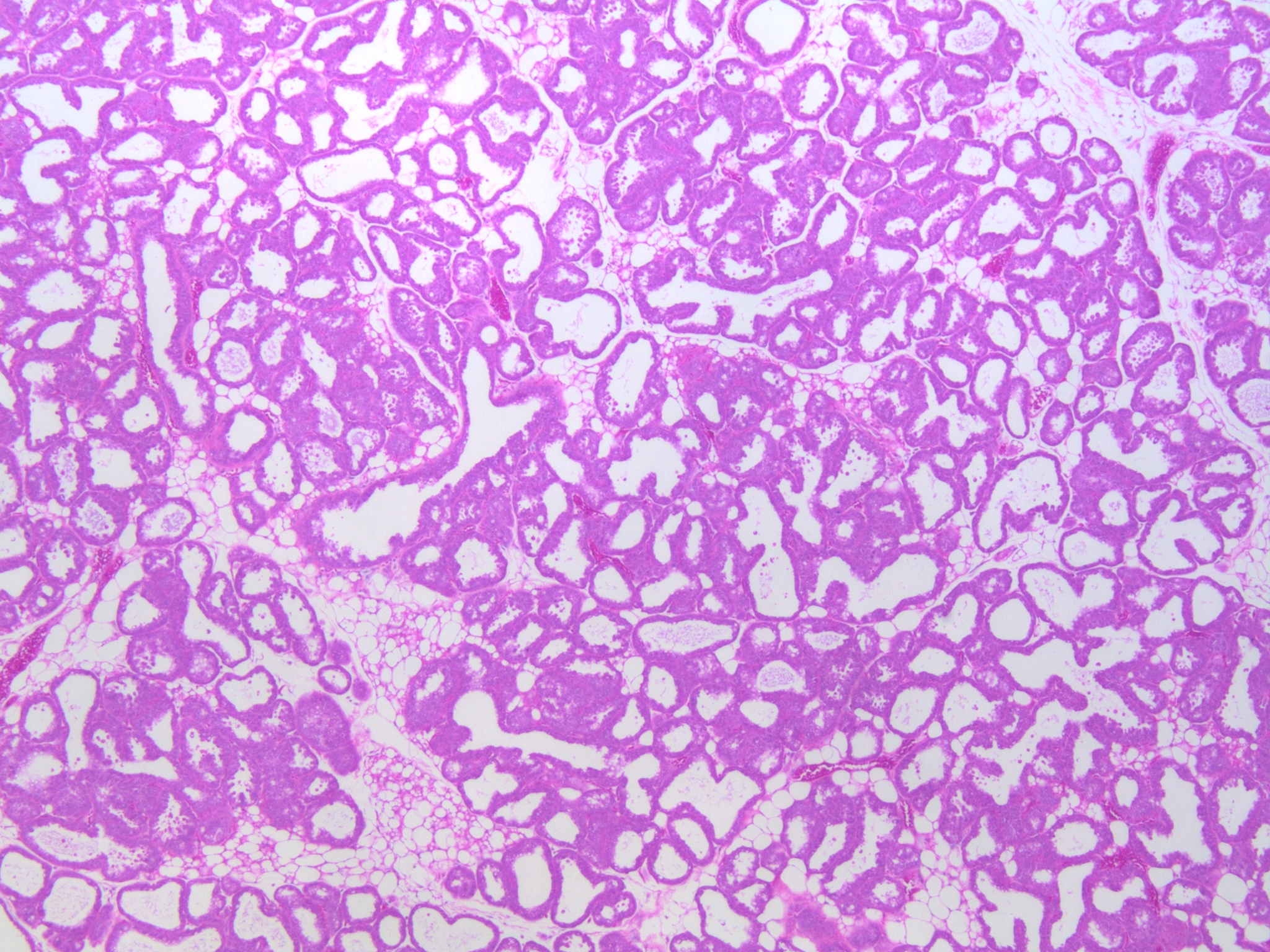

Supplement: Figure 6—source data 14. [file elife-78695-fig6-data14.zip › Figure 6--Source Data 14--5x_t952_2.tif]

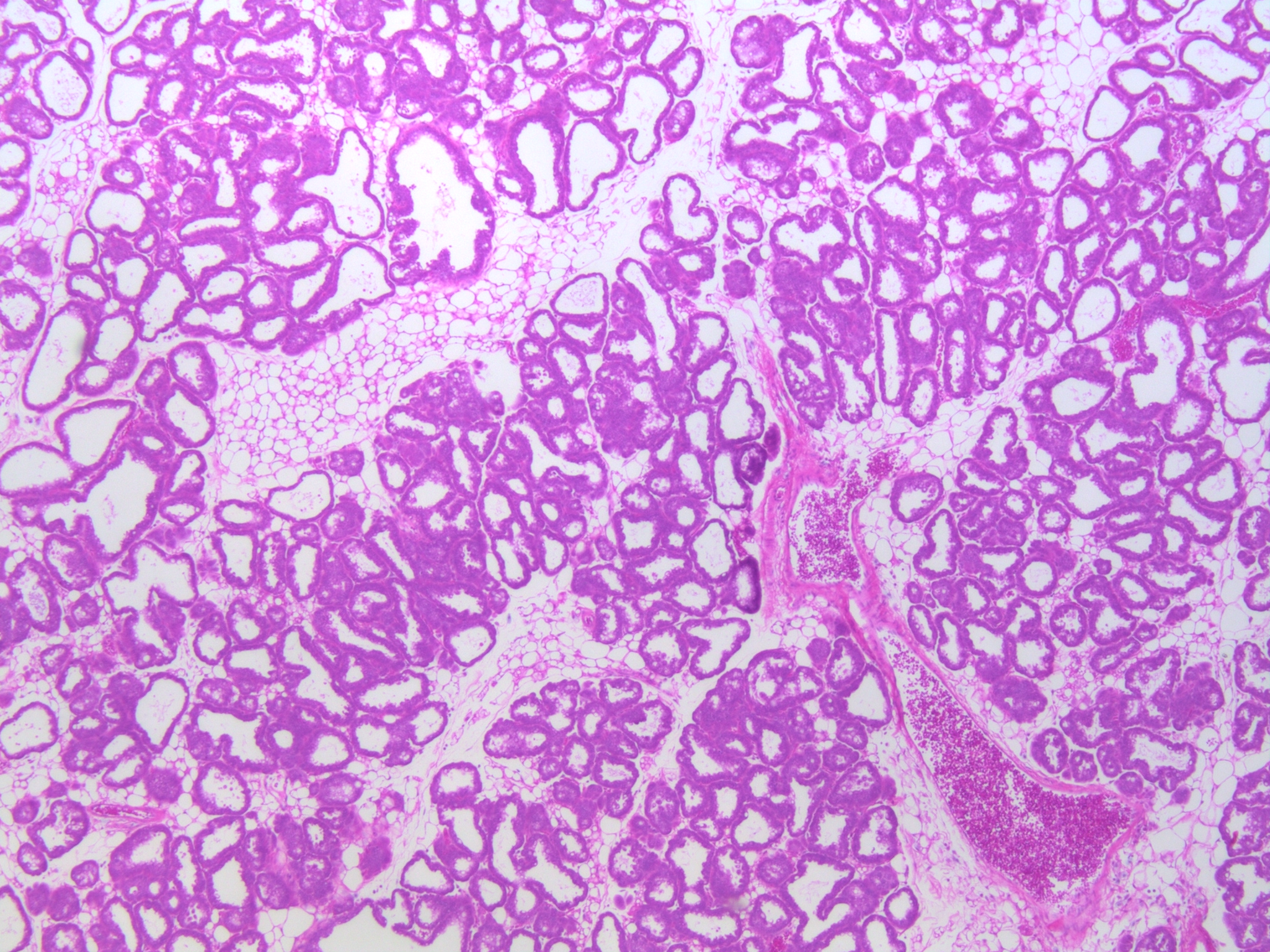

Supplement: Figure 6—source data 15. [file elife-78695-fig6-data15.zip › Figure 6--Source Data 15--5x_t646_3.tif]

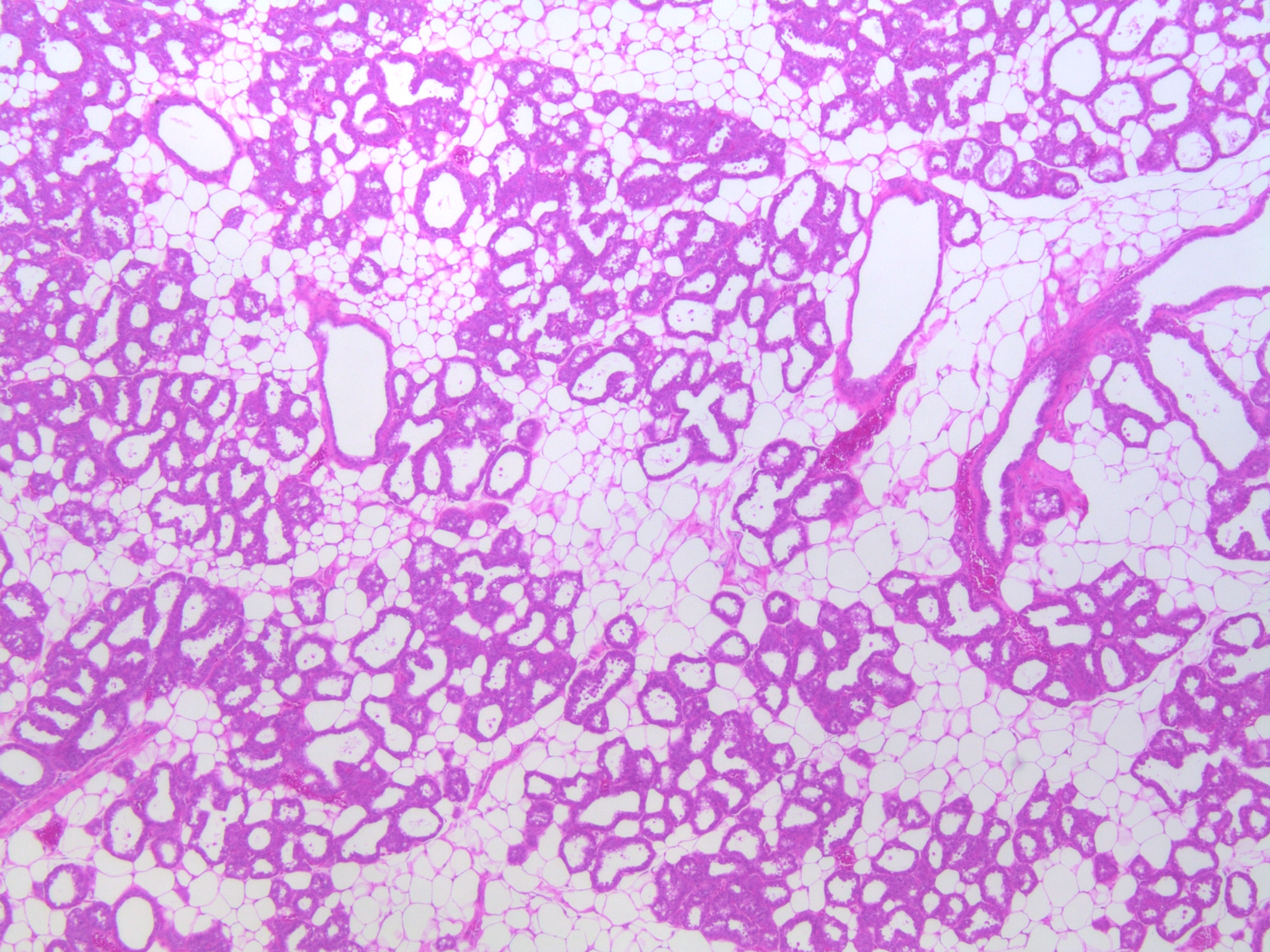

Supplement: Figure 6—source data 16. [file elife-78695-fig6-data16.zip › Figure 6--Source Data 16--5x_t561_02.tif]

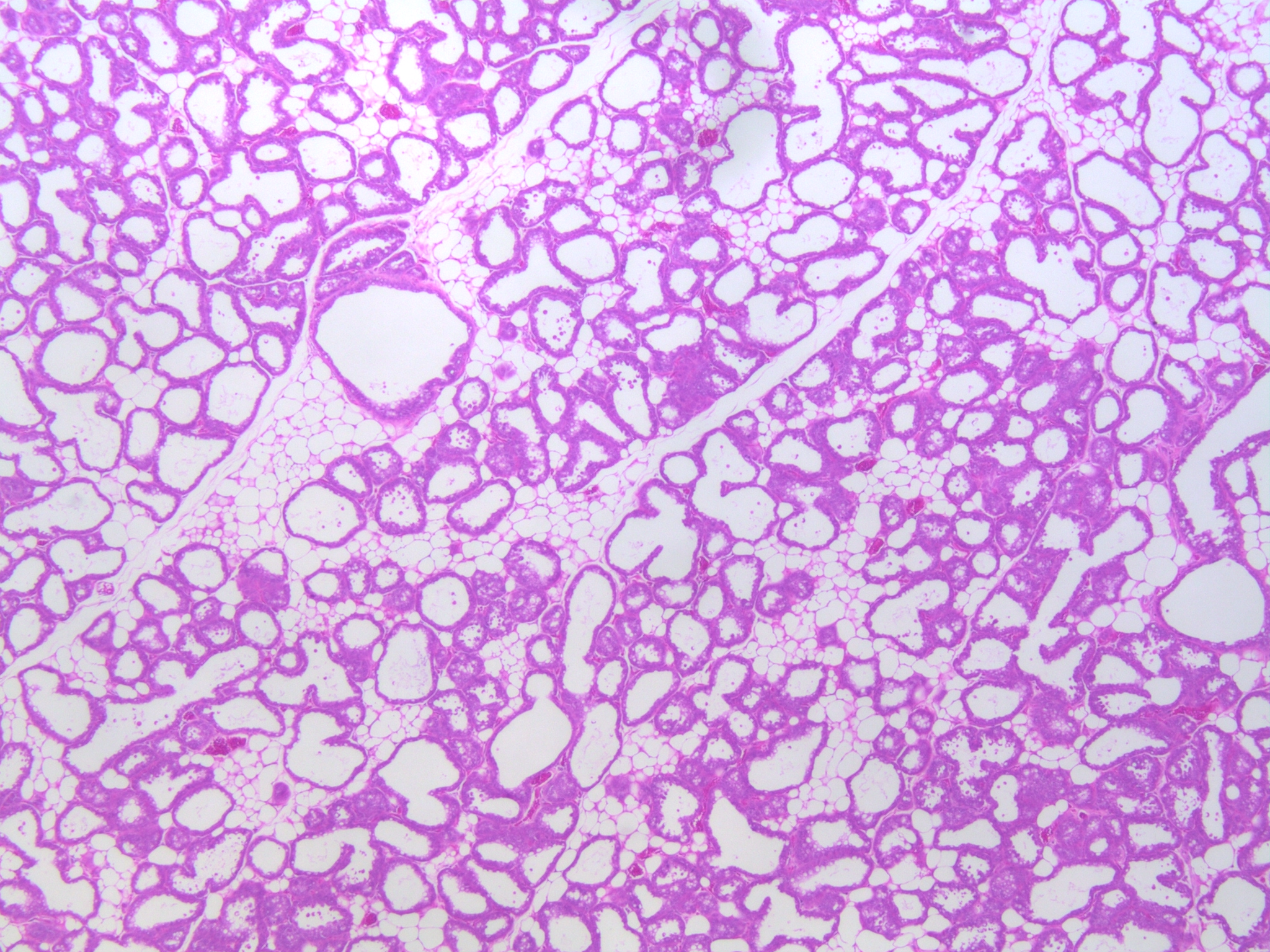

Supplement: Figure 6—source data 17. [file elife-78695-fig6-data17.zip › Figure 6--Source Data 17--5x_t909_.tif]

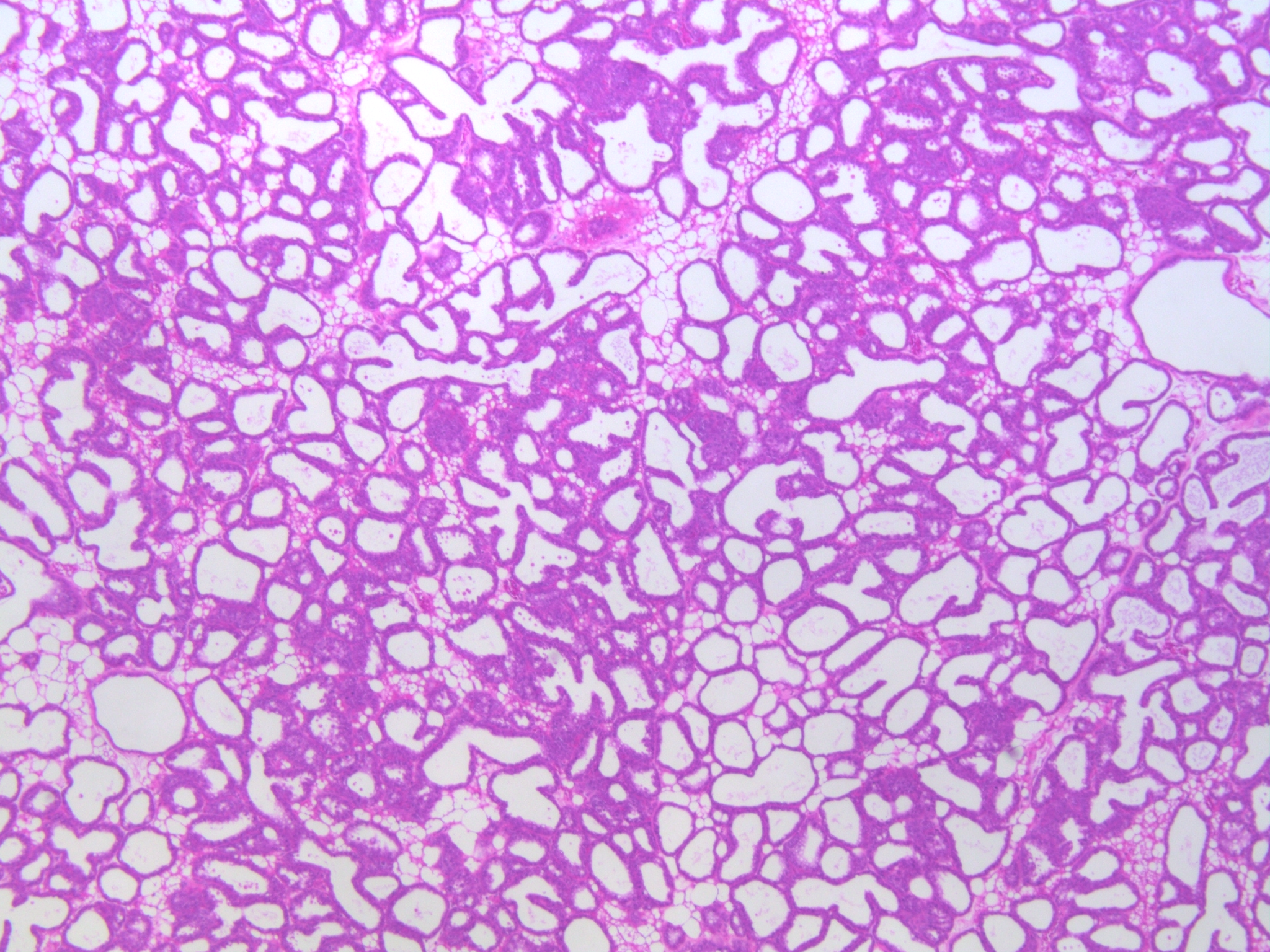

Supplement: Figure 6—source data 18. [file elife-78695-fig6-data18.zip › Figure 6--Source Data 18--5x_t771_03.tif]
